# Health first, convenience second: Caregiver perspectives of commercially produced complementary foods in five Southeast Asian capital cities

**DOI:** 10.1111/mcn.13600

**Published:** 2023-12-07

**Authors:** Helen Walls, Alissa Pries, Saipin Chotivichien, Phuong Huynh, Umi Fahmida, Jessica Blankenship

**Affiliations:** ^1^ London School of Hygiene & Tropical Medicine, Faculty of Public Health & Policy London UK; ^2^ Helen Keller International New York New York USA; ^3^ Department of Health Ministry of Public Health Bangkok Thailand; ^4^ National Institute of Nutrition Hanoi Vietnam; ^5^ Department of Nutrition, Faculty of Medicine Universitas Indonesia–Dr. Cipto Mangunkusumo General Hospital Depok Indonesia; ^6^ Southeast Asian Ministers of Education Organization Regional Centre for Food and Nutrition (SEAMEO RECFON) Pusat Kajian Gizi Regional Universitas Indonesia Jakarta Indonesia; ^7^ UNICEF East Asia and the Pacific Regional Office Bangkok Thailand

**Keywords:** child health, commercially produced complementary foods, complementary feeding, diets, Southeast Asia

## Abstract

Caregivers in low‐ and middle‐income countries increasingly feed commercially produced complementary foods (CPCF) to older infants and young children—shaped by factors including industry promotion. The dynamics of CPCF consumption and caregiver knowledge, attitudes and behaviours regarding complementary feeding practices are poorly understood in these settings. We examined how caregiver knowledge/attitudes/behaviours about CPCF shape the feeding of older infants and young children in the capital cities of five countries in Southeast Asia (Bangkok, Hanoi, Jakarta, Kuala Lumpur, Manila). An online, web‐based, cross‐sectional panel survey was conducted among mothers of a child aged 6−23 months. One hundred participants were included in each of the five capital cities. Questionnaires were undertaken in the official language of each city. Data were analysed in Stata (version 17.0), using *χ* ^2^ tests to examine difference between variables of interest. All mothers purchased CPCF for their youngest child aged 6−23 months at the time of survey. CPCF were commonly fed to children at least once per day, and in many of the five cities at most or every feeding. While factors such as convenience and affordability influenced CPCF purchase, mothers primarily purchased CPCF for nutritional reasons. The most common source of feeding information was health care professionals, followed by social media. CPCF are ubiquitous in the diets of older infants and young children of educated middle–upper socioeconomic status mothers in capital cities of Southeast Asia, with perceived healthiness a key driver in selecting CPCF. A strong governmental regulatory response to industry marketing/promotion will be critical to addressing CPCF appropriateness, including health and nutritional claim use.

## Key messages

- Commercially produced complementary foods (CPCF) are ubiquitous in the diets of older infants and young children of educated middle−upper socioeconomic status mothers in capital cities of Southeast Asia.
- While factors such as convenience and price and affordability influenced CPCF purchase, mothers in this study said they mainly purchased CPCF for perceived nutritional reasons.
- A strong government regulatory response to mandate CPCF standards for nutrient content, labelling and promotion will be critical in ensuring nutritional quality of CPCF and appropriate promotion.

## Graphical abstract

Commercially produced complementary foods (CPCF) are ubiquitous in the diets of older infants and young children of educated middle−upper socioeconomic status mothers in capital cities of Southeast Asia. While factors such as convenience and affordability influenced CPCF purchase, mothers primarily purchased CPCF for nutritional reasons.

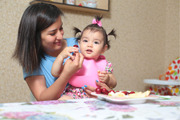

## 1 INTRODUCTION

The barriers to meeting older infant and young children's nutrient requirements are uniquely challenging, with the context for policy and programme interventions fast changing. In many low‐ and middle‐income country (LMIC) contexts, families are increasingly moving to cities, and diets are often constrained due to poverty, inequities and the increasing cost of nutritious food (Gaupholm et al., 2022; Vilar‐Compte et al., 2021). Concurrently, growing proportions of women are participating in the workforce, often with continued responsibility for most caregiving and household duties, restricting the time they have for preparing healthy homemade foods (Sharman et al., 2019, Tampah‐Naah et al., 2019). The changes to local food environments underlying the shifts in dietary patterns are characterized by the pervasive availability and aggressive marketing by food companies of processed foods in LMICs (Stuckler & Nestle, 2012). This includes commercially produced complementary foods (CPCF) targeted at older infants and young children (Baker et al., 2021; Karageuzian et al., 2021), some of which can be high in free sugars or sodium (Cogswell et al., 2015; Grammatikaki et al., 2021; Khosravi et al., 2023) making them inappropriate for young child feeding (Thompson, 2020). CPCF are now abundant in LMIC markets, including in countries of Southeast Asia (Bassetti et al., 2022; Green et al., 2019; Hinnouho et al., 2023; Pries et al., 2016). Consequently, millions of families worldwide are shifting from traditional diets towards diets characterized by greater quantities of convenient, packaged and often ultra‐processed foods high in salt, sugar and unhealthy fat, and low in essential nutrients (Unicef, 2019)—leading to increasing prevalence of obesity and risk of diet‐associated noncommunicable disease in children and adolescents (Neri et al., 2022).

Product marketing is a feature of the food environment that strongly influences consumer behaviour. As described by Karageuzian et al. (2021) its influence ‘mak[es] products more salient in consumers' minds, increasing product recall and recognition at the point of sale, creating positive associations and expectations and subsequently increasing purchase intention’. More broadly, promotional techniques also shape social norms regarding product acceptability and what is considered ‘normal’ to be consumed, including by children, in different life stages (Karageuzián et al., 2021).

Packaging is one strategy that food companies use to signal that their products are appropriate for children (Hawkes, 2010). With CPCF, packaging often includes cartoon characters, bright colours and cues to convey the idea that the products are healthy and specifically developed for children (Karageuzián et al., 2021).

Food companies are increasingly using digital marketing (also called online marketing, through use of the Internet and online digital technologies such as computers, mobile phones and other digital media and platforms) to sell CPCF. Parents and caregivers are the main targets of digital marketing campaigns, which include content related to the enjoyment and social aspects of food consumption, as well as the notion that these CPCF products are healthy and contribute to children's growth and development (Karageuzián et al., 2021).

CPCF consumption is highly prevalent in many LMIC contexts, replacing and often exceeding the consumption of homemade foods in some populations (Kavle et al., 2015; Maslin & Venter, 2017). Several studies in LMICs of Africa and Asia suggest that the purchase of CPCF is associated with higher socioeconomic status, with poorer families unable to afford such products (Abeshu et al., 2016; Cetthakrikul et al., 2022; Tampah‐Naah et al., 2019). However the converse has also been found—for example in Phnom Penh, Cambodia (Pries et al., 2017)—highlighting the complexity and context‐specific nature of CPCF use. Parental beliefs, values and perceived norms are a key influence on complementary feeding practices (Dattilo et al., 2020, Sharman et al., 2019). Studies of perceptions around CPCF use in LMIC contexts are limited, but insights can be drawn from research in high‐income country (HIC) contexts. Reasons reported by British caregivers for purchasing these products included convenience, promotion and food safety (Isaacs et al., 2022), American caregivers have reported health reasons, followed by taste and child's comfort (Afflerback et al., 2013), and Australian mothers have reported product acceptability and preferring organic CPCF (Begley et al., 2019). Food manufacturer's develop their marketing around these concerns—for example, emphasizing the healthiness, safety or organic nature of the foods—and this may undermine caregiver confidence, promote confusion about the optimal age for introducing solid foods and shift caregivers away from healthier homemade or less expensive foods (Dearlove et al., 2021).

Food product labels contain important information that influences consumer decision‐making, and global standards exist with regard to labelling CPCF products but these are rarely enforced. Increasingly, health advocacy organizations have called for warning labels on packaged foods, including for products targeting children (Popkin et al., 2021). In Chile, for example, such warnings have been implemented in the form of black octagonal stop signs with text describing the high critical nutrient (e.g., ‘high in sugar’) (Correa et al., 2019). Correa et al. (2019) found that this affected perceived healthiness of the products, with a suggestion of changes in social norms.

Despite growing interest among the public health community and national governments in regulating food products consumed by children, relatively little is known about the dynamics of CPCF use and the knowledge, attitudes and behaviours of caregivers shaping them, particularly in LMIC contexts. To address this knowledge gap and inform advocacy for regulation of CPCF, we undertook this study to understand caregiver knowledge, attitudes and behaviours in regard to CPCF in the capital cities of five countries in Southeast Asia. The study objectives were to understand: (1) CPCF purchasing and feeding dynamics; (2) caregiver motivations and reasons for purchasing CPCF; (3) how caregivers perceive nutrition/health claims and labelling information on CPCF; (4) and sources of information for caregivers on older infant and young child feeding.

## 2 METHODS

We undertook an online web‐based (accessed via smartphone, tablet or computer) survey, implemented between 30 June and 15 July 2022. The survey was enumerated by NielsenIQ, a leading consumer intelligence company, drawing from existing NielsenIQ survey panels (Nielsen, 2023) in each of the five capital cities (Jakarta, Indonesia; Kuala Lumpur, Malaysia; Manila, Philippines; Bangkok, Thailand; and Hanoi, Vietnam). From the NielsenIQ general population survey panels, mothers aged 18 years and over with a child 6−23 months of age were enroled in the survey, with oversampling of mothers with a child aged 6−18 months to achieve adequate numbers of mothers with children in this age group. Rolling admission was implemented until a total of 100 participants were obtained in each of the five capital cities.

Survey participants in each of the cities were compensated for participation. Nielsen 7provides data quality checks on survey participation and follows an established ‘double opt in consent methodology’. Firstly, potential participants provide consent to be a part of the NielsenIQ panel, to be approached for participation in studies suitable for their profiles and indicate agreement with privacy policy and other panel terms. A secondly consent for participation was obtained at the time of the survey for this specific CPCF study.

Mothers participating in this survey were directed to answer questions regarding their youngest child aged 6−23 months. The survey contained 25 questions with a median enumeration time of 7 min in Jakarta, Bangkok and Hanoi and 10 min in Kuala Lumpur and Manila. Questionnaires were written in the official language of each of the five countries (Bahasa Indonesian, Thai, Vietnamese, Malay and Tagalog, respectively). Questions and associated response types regarding CPCF consumption and associated labelling were developed by several of the coauthors and expert advisors to the study. They were accompanied by representative images of the types of CPCF and labelling available in each country to reduce recall bias and confusion with other packaged foods commonly fed to older infants and young children but not specifically labelled for children under 3 years of age. The questions addressed the four study objectives outlined above, as well as basic demographic information. See Supporting Information: Appendix 1 for the full questionnaire (English version).

A definition of CPCF was provided to mothers at the start of the survey, which was based on the definition used by the WHO but adapted to be understandable for survey participants (World Health Organization, 2019). It read as follows:

CPCF are products that are either packaged as ready‐to‐eat for the child in a jar, pouch, bag, box or other container, or prepared with the addition of liquids (cereals and porridges). Packaged complementary foods include full meals (such as [country relevant examples]), meal components (for example vegetable purees), desserts (baby custards) and snacks (like biscuits, puffs, fruit snacks). Packaged complementary foods are specifically labelled and marketed for young children. This does not include other commercially packaged foods that do not specify on their label they are for young children below 3 years of age, but which your children might commonly consume – such as biscuits, chips, sweets or commonly prepared foods purchased outside the home such as borbor, rice porridges, fresh fruit. This does not include infant formula or breastmilk substitutes.

The categories of CPCF assessed were: cereals and porridges, including baby oatmeal and rice porridge; purees (vegetable or fruit only, or both); dairy products, including baby cheeses and yoghurt but excluding beverages; foods/meals that contain meats, including eggs, fish, beef, chicken, pork and lamb; snacks/finger foods, including savoury snacks (puffs, rusks/teething biscuits, crackers, crisps, buns, savoury pastries), fruit snacks (fresh or dried fruit including freeze dried fruit) and sweet snacks (yoghurt fruit bites, fruit chews, jellies, gummies, sweet biscuits, cakes, sweet pastries, cereal bars, pudding, gelatin, candies, chocolate, jam, sweet spreads).

In the context of buying a CPCF product for the first time, mothers were asked how often they read the nutritional information (described as usually being provided in a box on the package, and including information on the fat, salt, sugar, protein and carbohydrate composition), if they refer to label claims for vitamin content (e.g., high in vitamin C, good source of vitamin A), and if they trust the vitamin content claimed on a label (e.g., that the product actually is high in vitamin C or vitamin A). The responses were categorized as ‘often’, ‘sometimes’, ‘rarely’ or ‘never’.

Mothers were also asked where they go for advice and information on feeding their children. Possible responses included doctors or health care professionals, mother or mother‐in‐law, other family members, friends, online parent groups, social media, parents'/mothers' group, online through web searches and other sources.

Data were received from Nielsen in Excel data files, and Stata version 17.0 (StataCorp LLC) was used to examine differences between prespecified variables of interest, using *χ* ^2^ tests.

## 3 RESULTS

### 3.1 Demographic information of survey participants

Table 1 presents demographic information for survey participants. In each city, most of the children (56%−73%) were aged 12−23 months. Mean maternal age was 31 years, and most mothers (over 80%) were aged 18−35 years, except for Hanoi, which had a higher proportion of mothers aged 36 years and older compared to the other four cities (*p* = 0.016). Most mothers had achieved higher education with either a vocational/bachelor's degree or higher (86%−97%), with a particularly high proportion (52%) of mothers from Kuala Lumpur having a postgraduate degree or higher compared to mothers from the other four cities (*p* < 0.001). Most mothers surveyed were of a ‘high’ socioeconomic status, particularly in Jakarta and Kuala Lumpur (70% and 81%, respectively). A higher proportion of mothers (14%) from Hanoi were of a ‘low’ socioeconomic status compared to the other cities, while two‐thirds of mothers from Manila were of a ‘medium’ socioeconomic status (*p* < 0.001). Median household size of the mothers was four for all cities except Manila, for which it was five.

**Table 1:** Demographic information of study participants in five major Southeast Asian cities, 2022.

		Bangkok (*n* = 100) (%)	Hanoi (*n* = 100) (%)	Jakarta (*n* = 100) (%)	Kuala Lumpur (*n* = 100) (%)	Manila (*n* = 100) (%)	*p* Value^a^
Mothers of a child aged (months), (%)	6−11	32	44	27	34	43	0.054
	12−23	68	56	73	66	57	
Mother's age (years), (%)	18−24	11	9	9	3	14	0.016
	25−35	74	59	72	77	74	
	36−45	12	23	17	18	10	
	46+	2	2	1	1	1	
	Not provided	1	7	1	1	1	
Highest level of education,^b^ (%)	High school or lower	3	2	14	6	6	<0.001
	Vocational/bachelors	91	87	80	42	89	
	Postgraduate degree or higher	6	8	6	52	5	
Socioeconomic level, (%)	High	58	55	70	81	30	<0.001
	Medium	36	31	26	15	67	
	Low	6	14	4	4	3	

*Note*: The SES variable was calculated using principle components analysis (PCA) using reported household income and asset data the classify households into six SES groups following NielsenIQ methodology.

^a^ *p* Values calculated from *χ* ^2^ tests.

^b^ Highest education level is categorized as high school or less (elementary school or lower, junior high school, high school), vocational/bachelors (vocational school, college/university bachelor's degree or equivalent), postgraduate degree or higher (master's degree, doctoral graduate).

### 3.2 Purchasing and consumption dynamics of CPCF

Mothers were asked questions about their purchasing and use of CPCF (data not shown unless specified). All mothers stated they currently purchase CPCF for their youngest child aged 6−23 months. In the past month, the prevalence of feeding CPCF ‘about once a day’ averaged 36% across the five cities, ranging from 29% in Bangkok to 39% in Jakarta (*p* = 0.058). Many mothers (34%−57%) had given their child CPCF at every or most feedings or meals. CPCF feeding frequency varied by child age and was higher for older children (>12 months) in all cities, a pattern that was less pronounced in Hanoi. Across all cities, CPCF were commonly fed to children *at least* once per day (66%−86%), and at most or every feeding/meal (34%−57%). However, mothers in Hanoi and Kuala Lumpur were the most likely to feed CPCF to their child at a lower frequency of ‘every few days’ (24% and 23%, respectively) (*p* = 0.013), and 14% of mothers in Kuala Lumpur reported feeding CPCF ‘rarely’.

CPCF were most commonly fed as a snack or between meals, although ranging considerably from 78% of the time in Kuala Lumpur to 97% in Jakarta (*p* = 0.001). The next common was feeding ‘when out of the house’, although ranging from 45% in Manila to 72% in Kuala Lumpur (*p* = 0.001), and ‘as full meal at home’, although ranging from 34% in Jakarta to 70% in Manila (*p* < 0.001).

As presented in Table 2, cereals and porridges were the most common type of CPCF in all cities except Hanoi, where the most common CPCF was dairy food products. Vegetable and fruit purees were also commonly fed in Bangkok, Kuala Lumpur and Manila, while snacks/finger foods were commonly fed in Hanoi and foods/meals that contain meat were common in Bangkok and Hanoi.

**Table 2:** Types of formulated commercial foods provided to children aged 6−23 months in five major Southeast Asian cities, 2022.^a^

	Bangkok (*n* = 100) (%)	Hanoi (*n* = 100) (%)	Jakarta (*n* = 100) (%)	Kuala Lumpur (*n* = 100) (%)	Manila (*n* = 100) (%)	*p* Values^b^
Cereals and porridges	89	74	91	90	93	<0.001
Purees (vegetable or fruit only)^c^	82	69	69	80	81	0.047
‐ Vegetable	72	61	58	68	68	0.044
‐ Fruit	67	65	67	80	77	0.001
‐ Both	91	86	74	91	81	0.002
Dairy products (excl. beverages)	80	83	59	69	70	0.001
Purees/meals that contain meats	75	75	63	55	37	<0.001
Snacks/finger foods^d^	62	79	65	42	40	<0.001
‐ Fruit	85	76	57	74	68	<0.001
‐ Savoury	97	81	75	81	83	<0.001
‐ Sweet	85	84	82	86	88	<0.001

^a^ The question asked to the caregiver being ‘What type of packaged complementary foods do you purchase for your child aged 6−23 months (under 2 years)?’

^b^ *p* Values calculated from *χ* ^2^ tests.

^c^ The figures below this main ‘purees’ category, for different types of purees (vegetable, fruit and ‘both’), are presented as a percentage of the main purees category.

^d^ The figures below this main ‘snacks’ category, for different types of snacks (fruit, savoury and sweet snacks), are presented as a percentage of the main snacks category.

The types of CPCF offered to children varied for younger (6−11 months) versus older (12−23 months) children although not markedly (*p* = 0.991 in Bangkok; *p* = 0.459 in Hanoi; *p* = 0.118 in Jakarta; *p* = 0.849 in Kuala Lumpur; *p* = 0.994 in Manila). In Bangkok, older children were more likely to receive all CPCF types compared to their younger counterparts (*p* = 0.991). In Hanoi, older children were more likely to receive cereals and porridges (43% vs. 31%), dairy food products (48% vs. 35%) and snacks/finger foods (48% vs. 31%). In Jakarta, older children were more likely than the younger children to receive purees (74% vs. 59%) and dairy food products (62% vs. 52%) (*p* = 0.118). In Kuala Lumpur, older children were more likely to receive dairy food products (74% vs. 59%) (*p* = 0.849). In Manila, older children were more likely to receive snack/finger foods (46% vs. 33%) (*p* = 0.944).

CPCFs were most often purchased in a supermarket in all cities, however, this ranged from 85% in Manila to 41% in Jakarta (*p* < 0.001). The next most common place of purchase was ‘baby store’ in Kuala Lumpur and Hanoi (13% and 33%, respectively, *p* < 0.001), and ‘mini mart’ in Jakarta and Bangkok (40% and 12%, respectively, *p* < 0.001).

### 3.3 Caregiver motivations and reasons for purchasing CPCF

**Figure 1:**
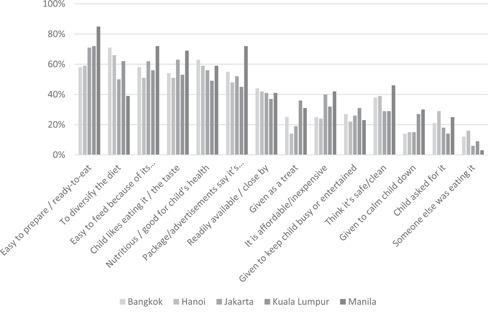
Reasons mothers purchased formulated commercial foods in the past month for children aged 6−23 months in five major Southeast Asian cities, 2022. The question asked to the caregiver being ‘What are your reasons for purchasing the packaged complementary food(s)?’

Figure 1 presents all the reasons mothers provided for purchasing CPCFs in the past month. The most frequently provided reason to purchase CPCFs in three of the cities (71% in Jakarta, 72% in Kuala Lumpur and 85% in Manila) was due to ease of preparation and the food being ready‐to‐eat. The most common reason to purchase CPCFs in the remaining two cities (71% in Bangkok, 66% in Hanoi) was to diversify the child's diet. Mothers were then asked to provide the *main* reason they purchased CPCFs. In all cities, one of the most common reasons for purchasing CPCF was related to nutrition and health benefits (the package/advertising say its nutritious/good or believing that the food is nutritious or good for the child's health and development). In Kuala Lumpur and Manila, the main reason was that the food was convenient (easy to prepare/ready to eat) (*p* = 0.004).

**Figure 2:**
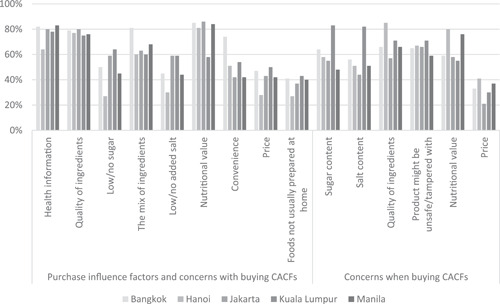
Purchase influence factors and concerns with buying formulated commercial foods for children aged 6−23 months in five major Southeast Asian cities, 2022. The questions asked to the caregiver for each subsection of the figure being ‘What factors influence your decision to purchase a packaged complementary food?’ and ‘What concerns do you have when buying packaged complementary foods for your child?’

Figure 2 presents information regarding the factors influencing mothers' purchase of CPCF (on the left) and their concerns with purchasing CPCF (on the right). The most common response to the influence on CPCF purchase was ‘nutritional value’ (81% to 86%), except for Kuala Lumpur (*p* < 0.001), where the most common influence was ‘health information’ (78%). ‘Quality of ingredients’ was also highly ranked as a motivation for purchase in several cities (75%−80%). The most common concern when purchasing CPCF in Jakarta was that the product might be unsafe or tampered with (66%), while ‘sugar content’ in Kuala Lumpur (83%), ‘nutritional value’ in Manila (76%) and ‘quality of ingredients’ in Bangkok and Hanoi (66% and 85%, respectively) were also reported.

### 3.4 Caregiver perceptions of nutrition/health claims and labelling in purchasing decisions for CPCF

Across the five capital cities, most mothers said that they ‘often’ or ‘sometimes’ read the nutritional information (97% or more in all cities), with ‘often’ ranging from 71% in Hanoi, 73% in Bangkok, 80% in Kuala Lumpur, 87% in Jakarta, to 90% in Manila (*p* = 0.002). Similarly, most mothers said that they ‘often’ or ‘sometimes’ refer to the label claims about vitamin content (98% or more in all cities), with ‘often’ ranging from 76% in Bangkok to 92% in Manila (*p* = 0.029). Most mothers also ‘often’ or ‘sometimes’ trust the nutrient/health claims made on a label, but the ‘often’ response was lower than for the previous two questions, ranging from 45% in Bangkok, 57% in Hanoi, 66% in Jakarta, 70% in Manila, to 74% in Kuala Lumpur (*p* < 0.001).

When purchasing CPCF, mothers in each of the capital cities most commonly reported looking at the ‘nutritional information’ (that could be anywhere on the label) (83%−90%). This was followed by looking at claims about health and nutrition benefits, and the list of ingredients. Images or design (31%−60%) were the least common label features looked at by mothers, followed by the name or brand (51%−72%).

Across younger and older age groups, the terms mothers reported to most likely influence their purchase of CPCF were ‘natural’ in Bangkok, ‘fortified’ in Hanoi, ‘natural’ and ‘low sugar’ in Jakarta, ‘no artificial ingredients’, ‘natural’ and ‘no artificial colours’ in Kuala Lumpur and ‘fortified’ in Manila. ‘Healthy’ was an important consideration across all the five cities (Table 3).

**Table 3:** Terms that influence formulated commercial food purchase for children aged 6−23 months in five major Southeast Asian cities, 2022.^a^

	Bangkok (*n* = 100)			Hanoi (*n* = 100)			Jakarta (*n* = 100)			Kuala Lumpur (*n* = 100)			Manila (*n* = 100)			*p* Value^b^		
	6−11 m	12−23 m	All	6−11 m	12−23 m	All	6−11 m	12−23 m	All	6−11 m	12−23 m	All	6−11 m	12−23 m	All	6−11 m	12−23 m	All
No artificial ingredients	53%	69%	68%	36%	52%	45%	56%	41%	45%	82%	85%	84%	63%	75%	70%	0.043	<0.001	<0.001
Natural	75%	79%	78%	61%	68%	65%	78%	66%	69%	91%	80%	84%	67%	74%	71%	0.516	0.103	0.019
No artificial colours	47%	68%	61%	50%	66%	59%	74%	56%	61%	76%	79%	78%	51%	58%	55%	0.242	0.272	0.01
No added sugar	50%	54%	53%	32%	21%	26%	48%	59%	56%	74%	79%	77%	30%	37%	34%	0.109	<0.001	<0.001
Low sugar	44%	62%	56%	43%	36%	39%	63%	64%	64%	71%	74%	73%	49%	54%	52%	0.438	<0.001	<0.001
Healthy	75%	78%	77%	59%	77%	69%	89%	74%	78%	62%	76%	71%	77%	81%	79%	0.372	0.482	0.369
Low sodium/salt	44%	68%	60%	48%	43%	45%	37%	60%	54%	53%	74%	67%	47%	58%	53%	0.198	0.001	0.027
Organic	69%	57%	61%	57%	63%	60%	63%	58%	59%	68%	62%	64%	74%	65%	69%	0.162	0.847	0.594
Fortified	66%	69%	68%	70%	75%	73%	19%	27%	25%	41%	53%	49%	84%	79%	81%	<0.001	<0.001	<0.001

^a^ The question asked to the caregiver being ‘Which of the following terms, if you saw them on the front label of a packaged complementary food would make you more likely to purchase that product for your child compared to a similar product without those terms?’

^b^ *p* Values calculated from *χ* ^2^ tests and refer to country comparisons.

### 3.5 Sources of information for caregivers on infant and young child feeding

Most mothers in all countries (75%−94%) said they go to a doctor or other health care professional for advice and information on feeding their children. Social media was generally the second most common source for mothers from all cities, but was most common in Hanoi (84%). Other sources included parent or mother groups, online parent groups, friends, mother or mother‐in‐law, other family members and online web searches.

## 4 DISCUSSION

CPCF use for older infant and young child feeding was highly prevalent among this sample of educated and largely middle and upper socioeconomic status mothers in five capital cities of Southeast Asia. All mothers surveyed reported purchasing CPCF at the time of the study. Overall, more than one‐third of mothers gave CPCF to their 6−23 month old child at least once a day, and almost half of mothers fed their child CPCFs at every or most feedings or meals. Perception of nutritional benefit was the most common influence on caregiver purchase of CPCF, with nutrition information on CPCF products commonly considered.

Previous data (Euromonitor International, 2018) suggests that CPCF comprises a rapidly growing but, as of 2018, still relatively small percentage share of the baby food market in the Southeast Asian region. Thus, our findings of highly prevalent use of CPCF suggest that compared with the region as a whole, consumption of CPCF is much higher in Southeast Asian capital cities, and amongst higher socioeconomic groups. They may also point to rapid growth of CPCF in this market since 2018, particularly in urban regions that tend to lead such nutritional transitions (Popkin et al., 2012; Ren et al., 2021).

The most common type of CPCFs provided to children across most of the cities were cereals and porridges, although in Hanoi it was dairy food products. Other commonly provided CPCF were vegetable and fruit purees, snack foods and foods or meals containing meat. These findings differ to those of a study in Uruguay, which reported that dairy products (milk desserts and yogurts) were the most prevalent CPCF consumed, followed by juices and soya‐based beverages (Karageuzián et al., 2021). These types of differences likely highlight the contextual nature of CPCF choice as influenced by aspects of culture as well as promotion. In our study, we found between‐city differences between types of CPCF fed to older versus younger children, with suggestion of greater frequencies being fed to children of the older age group than the younger children. These findings fit with those of HIC settings, such as in the United Kingdom, where in 2011 the prevalence of older infants and young children consuming CPCF rose with age (Department of Health & Food Standards Agency, 2011), and indicate that the types of CPCF used for young child feeding likely evolve as children move into different stages of textures/flavours. However we did find that older children (12−23 months) in Jakarta and Hanoi were more likely to eat CPCF intended for younger children (cereals and purees) than the younger children (6−11 months) for whom these products are intended.

The mothers in our study reported purchasing CPCF primarily for health reasons, considering CPCF nutritious or good for the child's health. They commonly reported reading and referring to nutritional information—information that could be anywhere on the label, rather than relating specifically to the nutritional information panel—on the products and reported often or sometimes trusting the nutritional/health claims. The convenience of CPCF was also highly influential in shaping their purchase, in keeping with considerable evidence globally of the influence of the convenient packaged nature of ultra‐processed foods in shaping their purchase (Machado et al., 2017; Reardon et al., 2021).

These findings regarding caregivers' strong consideration of product healthiness and the influence of nutrient/health claims on CPCF purchase is supported by considerable literature on how health/nutrient claims can create a ‘health halo’ for consumers. Such ‘health haloes’ can result in overestimation of product healthfulness, enabling their overconsumption (Chandon & Wansink, 2007; Jurkenbeck et al., 2022). Findings from a range of other (both HIC/LMIC) settings have also found CPCF to often be fed due to perceptions of healthiness (Dearlove et al., 2021; Maslin & Venter, 2017; Wrottesley et al., 2021) and is concerning given that most CPCF products in Southeast Asia have been nutrient profiled as nutritionally unsuitable for promotion for older infants and young children. Bassetti et al. (2022) examined the nutritional suitability of CPCF marketed in areas of Cambodia, Indonesia and the Philippines, and found that 96% of products in Cambodia, 90% of products in Indonesia and 63% of products in the Philippines were not nutritionally suitable for promotion for older infants and young children. Some specific CPCF types such as yoghurts, which range from healthy plain, unsweetened yoghurt to flavoured and containing high sugar levels, are more problematic to capture accurately in terms of health impacts. Devenish et al. (2019) describe how different types of children's yoghurt pouches located side‐by‐side on supermarket shelves sometimes contain ‘three‐to‐four‐fold differences in sugar content for the same flavour across different brands’.

The food environment, which is made up of food availability, price and product characteristics, including product healthiness or unhealthiness, has been described as the ‘interface’ between the individual and the wider food system (Turner et al., 2018). Whilst knowledge, attitudes and behaviours of individual caregivers is the ‘proximal’ factor shaping older infants and young children's diets, the convergence of different political, economic and social factors/trends strongly shape caregiver preferences and the local food environments in which individual caregivers make foods choices for them. These factors and trends include corporate supply and marketing of unhealthy products. Karageuzián et al. (2021) describe how ‘the food environment, characterized by the ubiquitous availability and marketing of ultra‐processed products, is one of the factors underlying which food caregivers choose for [older] infants [and young children]’. The authors found that whilst most CPCF products in Uruguay were excessive in sugar, they were packaged to suggest that they were appropriate for children, including for health reasons. Consistent with the results from previous studies, these elements included the use of ‘cartoon characters, bright colours and childish font’ as well as pictures of fruits/vegetables, claims related to the absence of particular ingredients (e.g., preservatives) and nutrient content claims, such as references to vitamins and minerals. Prior research has shown that consumers frequently rely on such aspects of food packaging to judge product healthiness rather than more complex information about product nutritional composition (Lähteenmäki, 2013, Machín et al., 2020). Thus, and as we also found in our study, caregiver perception of products' healthfulness is a leading factor shaping CPCF purchase and feeding practises. Increasingly too, digital marketing is shaping caregiver behaviour in regard to CPCF feeding (Dearlove et al., 2021) through its ability to provide seemingly tailored content—responding to caregiver desire for factual education appropriate to their personal infant feeding choices, and education sensitively provided using a nonjudgmental approach (Dattilo et al., 2020). Together, these findings are further evidence of the critical need identified in many studies of contexts globally to address corporate influence on food choice and nutrition (Baker et al., 2020; Milsom et al., 2020; Wood et al., 2021), including here in regard to the diets of older infants and young children.

In this study, although we did not directly assess the nutrient composition of the CPCF products identified, recent studies have assessed the nutrient composition of CPCF cereals, purees/meals and finger foods/snacks available in seven Southeast Asian countries and found concerning levels of added sugars, total sugar and sodium among a substantial proportion of products (Bassetti, Blankenship, White, Mulder, et al., 2023; Bassetti, Blankenship, White, Sweet, et al., 2023; Pries et al., 2023). The influence of food environments, including packaging and labelling practices, on individual caregiver behaviour highlights the need for governmental regulatory action to address rising consumption of potentially unhealthy CPCF products. This is also in the context of these products replacing aspects of local diets including healthier home‐prepared foods and reducing dietary diversity, as for example shown by Diana et al. (2017) in regard to older infants and young children in rural Indonesia.

Regulatory action could include measures such as developing and implementing strict standards on food labelling practices to ensure clear and accurate information is provided to prevent deceptive nutrient content claims on products unsuitable for older infants and young children, including providing warning labels on packaged foods high in specific nutrients (sugar, fat and sodium). Stricter nutritional composition standards, for example a ban on added sugars/nonsugar sweeteners and on the production of sugar‐sweetened beverages intended for older infants and young children, and limits on the use of pureed fruit and total sugar content of CPCF has also been suggested (Alexy et al., 2022). With labelling, evidence suggests that ‘the simpler the message, the higher the impact of consumer's behaviour’ (Correa et al., 2019), so simple front‐of‐package nutritional information could be an important tool. Bassetti, Khosravi, et al. (2023) have evaluated front‐of‐pack nutrition labelling in regard to CPCF. Importantly, strict regulation of digital marketing of CPCF products is also needed, given the increasing use of this practice by food companies (Dearlove et al., 2021; Karageuzián et al., 2021). In terms of addressing corporate influence on diets more systemically, Wood et al. (2023) have identified strategies that health advocates may take to counteract the challenging power dynamics involved. This includes specific actions to disperse concentrated corporate wealth and power, to strengthen countervailing power structures, to democratize corporate decisions‐making, to reform and democratize the global governance of corporations and to dissolve excessive and harmful corporate power. In terms of controlling the marketing of foods and beverages targeted toward infants and young children, we also recommend referring to the WHO's *Guidance on ending the inappropriate promotion of foods for infants and young children: Implementation Manual* (World Health Organization, 2017). There is a growing market in Southeast Asia for CFCP in the context of a triple burden of malnutrition in the region, thus targeted advocacy could be highly influential in improving the labelling of CPCF products and nutrition‐related health.

This study has several limitations. Firstly, our sample of 500 mothers residing in the capital cities of five Southeast Asian countries was not randomly selected and it is not nationally representative or representative of mothers residing in the cities where the study was done. Respondents were well‐educated mothers with internet access. Secondly, our methods did not include a standard dietary assessment and therefore we cannot conclude anything about the role of CPCF in infants and young children diets as a whole. Nevertheless, our findings suggest that CPCF are ubiquitous in older infant and young children's diets in this demographic of Southeast Asia. Thirdly, we had no way to validate mothers' responses and social desirability bias may have influenced the answers provided by mothers. Misreporting of food consumption, for example, is a common problem when asking respondents to recall foods that have been eaten. Evidence suggests that some foods and nutrients may be under‐ or over‐reported to a greater extent than others, but there is no information available on the level to which CPCF purchase and consumption is misreported in surveys. Fourthly, the responses provided often raise further questions that could be answered by further (often, qualitative) research.

Our study findings highlight the ubiquitous nature of CPCF in the diets of older infants and young children of educated (to higher education levels), middle and upper socioeconomic status mothers in five capital cities of Southeast Asia. It also highlights that perceived healthiness is a critical driver of the mothers' use of these products when feeding their young children. The importance of a strong governmental regulatory response, through development of standards to regulate the nutrient content, labelling and promotion of CPCF is critical, both in the Southeast Asian region and elsewhere, in safeguarding the diets of older infants and young children. Further research, particularly of a qualitative nature, could explore in greater detail the knowledge, attitudes and behaviours of caregivers regarding the foods provided to their older infants and young children, and how this is shaped by local food environments and the wider system of political, economic and social factors—including the role of industry and social media in this. Such research could also explore urban/rural differences and the influence of caregiver socioeconomic status and education on these relationships. This type of research is needed in a range of settings, including in Southeast Asia, given the context‐specific aspects of CPCF purchase and feeding dynamics. Working in tandem with those addressing other aspects of the commercial determinants of health, we also wish to emphasize the importance of a research focus on advancing the suite of strategies that health advocates can use—and action itself—to counteract the influence of corporate activity on food choice and nutrition.

## AUTHOR CONTRIBUTIONS

A. Pries and J. Blankenship conceptualized the study. H. Walls, A. Pries and J. Blankenship designed the study. H. Walls led the analysis of data received from NielsenIQ. H. Walls led the interpretation of results and drafting of the manuscript with input from A. Pries, S. Chotivichien, P. Huynh, U. Fahmida and J. Blankenship.

## CONFLICT OF INTEREST STATEMENT

The authors declare no conflict of interest.

## ETHICS STATEMENT

All information collected was anonymized by Nielsen with only the anonymized information used for analysis. NielsenIQ is a registered business in all five locations and follows the requisite guidelines to conduct surveys with panelists globally. The survey participants were all aged 18 years of age and above and are part of the general population. Protocols for the protection of human participants in this study were assessed through a research ethics review by the Health Media Labs (HML) Institutional Review Board (IRB) in April 2022. HML IRB is authorized by the United States Department of Health and Human Services, Office of Human Research Protections (IRB #1211, IORG #850, FWA #1102). This study's human participants' protection protocols received ethics review approval on 21 April 2022.

## Supporting information

Supporting information.

## Data Availability

The data that support the findings of this study are available on request from the corresponding author. The data are not publicly available due to privacy or ethical restrictions. Data relating to this work but not included in the final publication are available upon request. The code used for analysis is available upon request.
