# Supplementary material for: Health first, convenience second: Caregiver perspectives of commercially produced complementary foods in five Southeast Asian capital cities
Source: Matern Child Nutr. 2023 Dec 7;19(Suppl 2):e13600. doi: 10.1111/mcn.13600 (PMC10719061; doi:10.1111/mcn.13600)
Supplement: Supplementary file 1 — Supporting information. [file MCN-19-e13600-s001.docx]

**COMMIT Concept Note**

**The Southeast Asia Consortium for Improving Complementary Foods**

**Consumer perspectives on Commercially Available Complementary Foods (CACFs)**

**Overview**

COMMIT is a consortium of likeminded organizations working together to measure the quality of CACFs.^[[1]](#footnote-2)^ COMMIT is doing this by assessing and benchmarking the nutrient and labelling quality of CACF’s sold and marketed in Lao PDR, Cambodia, Vietnam, Thailand, Indonesia, Malaysia and the Philippines using the WHO Europe Nutrient Profile Model for Commercially Available Complementary Foods. This information will be used together with research on policies and legal instruments to advocate for better standards and legal measures to regulate the marketing of CACFs. In addition to the content and labelling analysis in the WHO Europe Nutrient Profile Model, COMMIT is collecting information on the micronutrient content of all CACFs assessed in each country to guide the development of micronutrient standards into the existing Nutrient Profile Model for Complementary Foods. Those micronutrient standards – if meeting all standards of the existing WHO Europe Nutrient Profile Model for Commercially Available Complementary Foods– would mean a CACF would be deemed ‘appropriate for marketing to children’. The micronutrient standards, plus the modified Nutrient Profile Model, would together form a Southeast Asia Nutrient Profile Model for Commercially Available Complementary Foods intended for use and adoption/adaptation in Southeast Asian countries.

**Current COMMIT research activities**

COMMIT is currently in the process of collecting data on:

1. The quality of CACFs in 7 countries including
   1. Nutrition content
   2. Labelling information
2. Price, availability and product categories
3. Existing legal and policy measures relating to Breast Milk Substitutes and CACFs in selected Southeast Asian Countries
4. Nutrient content analysis of sub-sample of CACFs

**Rationale for Data Collection**

To complement the information on quality of CACFs and the policy environment that influences their sale, composition and marketing in Southeast Asia, COMMIT is proposing to collect data from caregivers in Thailand, Vietnam, Indonesia, Malaysia and the Philippines on preferences, habits, decision making, and knowledge regarding CACFs. This information is a critical component of understanding where and why caregivers purchase CACFs, when and how frequently they use them, and what influences their purchasing decisions. Available market data shows that the market for CACFs is growing, but there is limited detail on use of the full range of CACF products and how they are perceived by caregivers.

This data will inform COMMIT’s advocacy and communications strategy and help COMMIT and stakeholders – including government and consumer groups – advocate for reformulation, regulation and better standards for CACFs.

**Survey Objectives**

1. Understand the purchasing and consumption dynamics of CACFs in 5 countries – type of data includes frequency, place of purchases, types of products purchased, and use of products
2. Understand caregiver motivations and reasons for purchasing CACFs in the 5 countries
3. Understand how caregivers perceive nutrition and health claims and other labels in purchasing decisions for CACFs in the 5 countries
4. Understand sources of information for caregivers on infant and young child feeding in the 5 countries

**Implementation**

The survey will be conducted via a web-based survey in Jakarta, Indonesia; Kuala Lumpur, Malaysia; Manila, Philippines; Hanoi, Vietnam; and Bangkok, Thailand, targeting at least 100 respondents per country. **The target respondent will be mothers of children 6-23 months**. The survey will be implemented by Nielsen, who will be responsible for translation, data collection, analysis, and reporting. The draft questionnaire is included on page 3.

**Methodology:** Web-based survey of mothers with children 6-23 months old.

**Sample Size:** Estimated 150 -250 Respondents per country. Minimum sample size of 100 caregivers per country who purchase CACFs (i.e. 100 caregivers complete the full survey), and 100 participants per country who meet the age criteria, but do not currently purchase packaged complementary foods.

**Recruitment**: Survey participants will be drawn from NielsenIQ’s panel participants.

**Working definition of Packaged complementary food (definition to be finalized):** Packaged complementary foods are products that are either packaged as ready-to-eat for the child in a jar, pouch, bag, box or other container, or prepared with the addition of liquids (cereals and porridges). Packaged complementary foods include full meals (such as ,…), meal components (for example vegetable purees), desserts (baby custards) and snacks (like biscuits, puffs, fruit snacks). Packaged complementary foods are specifically labelled and marketed for young children. This **does not include** other commercially packaged foods that do not specify on their label they are for young children below 3 years of age, but which your children might commonly consume – such as biscuits, chips, sweets or commonly prepared foods purchased outside the home such as borbor, rice porridges, fresh fruit.^[[2]](#footnote-3)^ **This does not include infant formula or breastmilk substitutes.**

## Draft Survey Tool

**Instructions for Participant**:

## **PN: START HERE**

This survey is designed to capture information about the purchase and use of **Packaged Complementary Foods** that are sold and marketed for children under the age of 3. You may refer know of them as ‘**baby foods’** or **‘toddler foods’**. Specifically, these foods are products that are either packaged as ready-to-eat for the child in a jar, pouch, bag, box or other container, or prepared with the addition of water or milk or other liquids. Packaged complementary foods include **cereals** (like cerelac, or baby rice), **full meals** (such as pureed chicken and rice), **meal components** (for example vegetable purees, fruit purees), **desserts** (baby custards) and **snacks** (like biscuits, puffs, fruit snacks). Packaged complementary foods are specifically labelled and marketed for children up to the age of 3.

[INSERT IMAGES PER COUNTRY HERE]

For this survey we are **not asking** about other commercially packaged foods that do not specify on their label they are for young children below 3 years of age, but which your children might commonly consume – such as biscuits, chips, sweets or commonly prepared foods purchased outside the home such as borbor, rice porridges, and fresh fruit.^[[3]](#footnote-4)^For the purpose of this survey we are **NOT asking you about infant formula or breastmilk substitutes.**

The information collected through this survey will be used by UNICEF in conjunction with research on policies and legal instruments to advocate for better standards and legal measures to regulate the marketing of CACFs. Your responses to all questions are strictly confidential and will be used for research purposes only. Participation is voluntary, and you may choose to withdraw anytime without consequences

[INSERT IMAGES PER COUNTRY]

Indonesia:


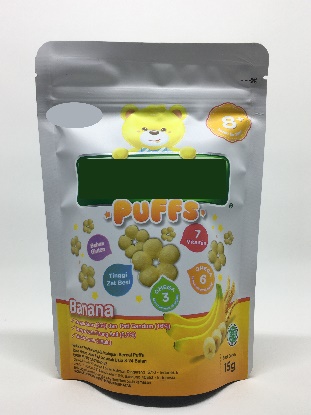

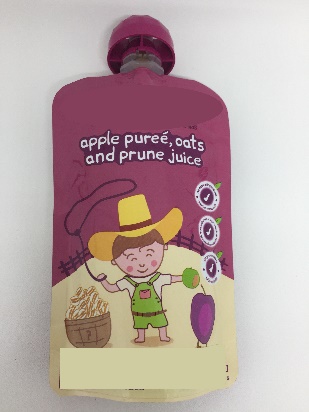

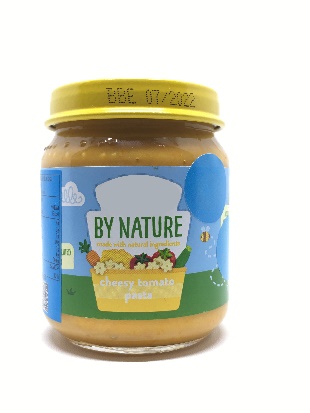

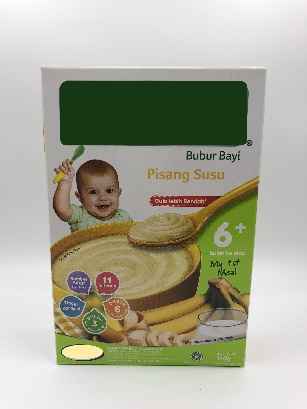


Malaysia
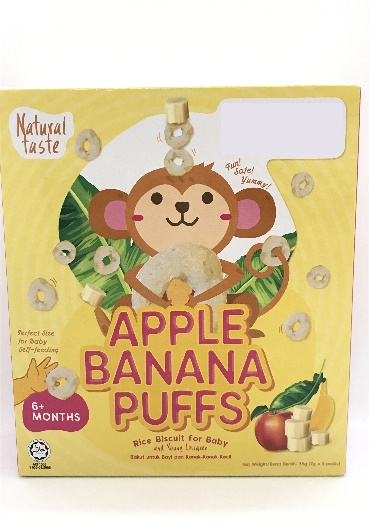

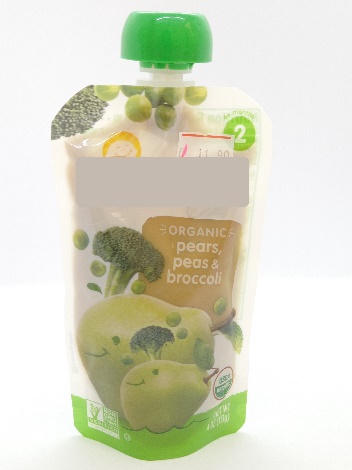

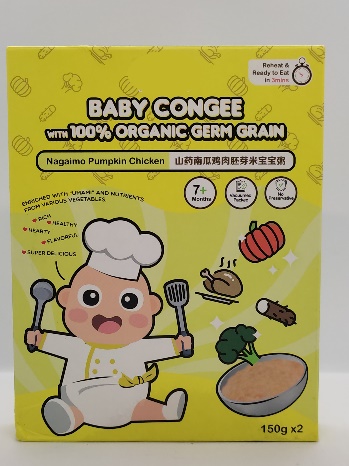

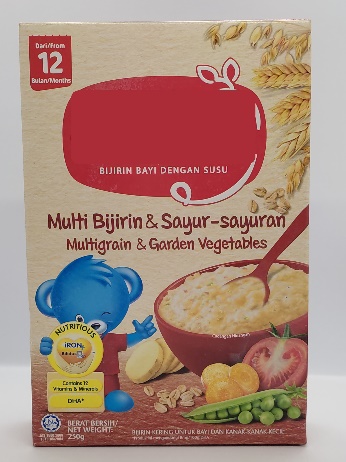


Philippines


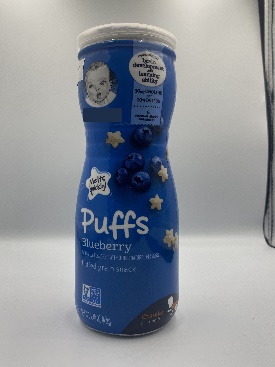

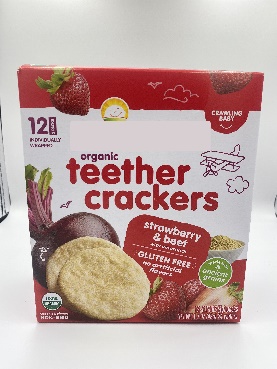

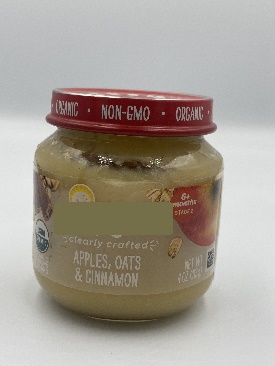

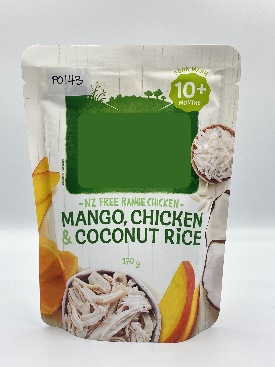

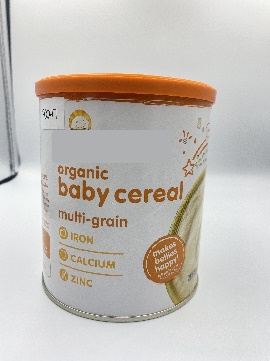


Thailand


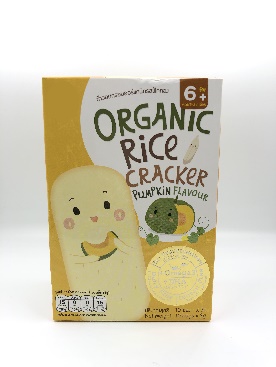

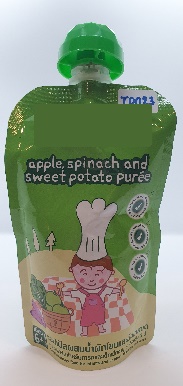

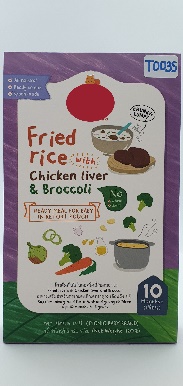

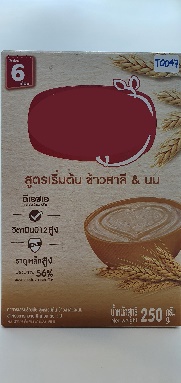

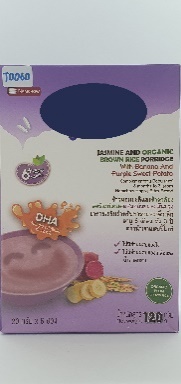


Vietnam


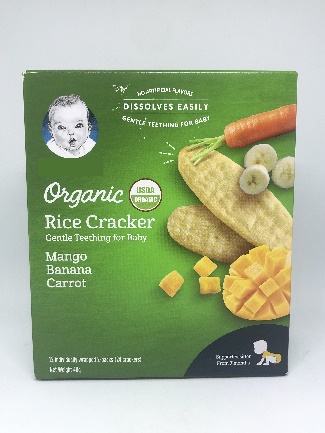

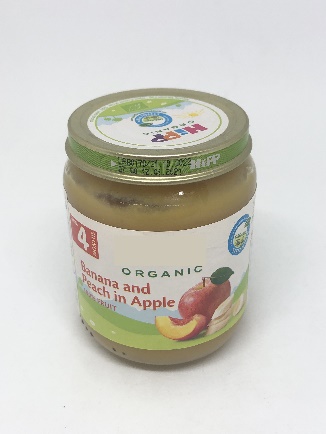

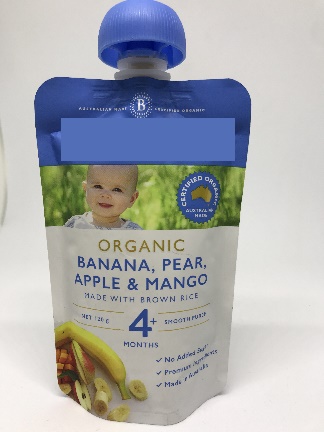

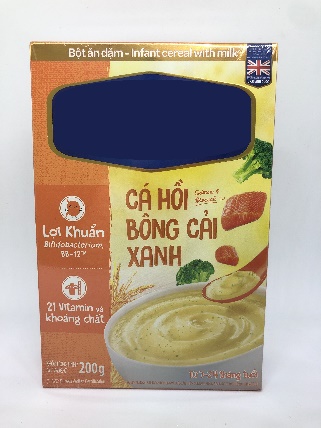

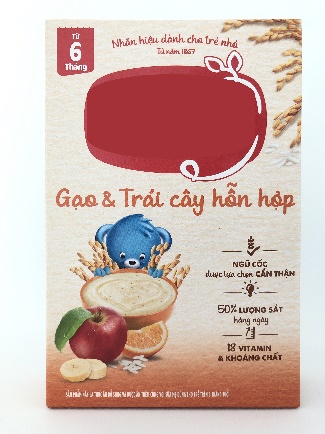


Do you agree to participate in this survey about on Commercially Available Complementary Foods (CACFs). ?

1. Yes – PN: CONTINUE
2. No - PN : CLOSE THE SURVEY

**[ PN- NEXT SCREEN]**

Thank you for taking the time out of your day to participate in this survey on Commercially Available Complementary Foods (CACFs).

Please take your time to read all of the information and answer each question as honestly and accurately as possible. The entire survey should take roughly 20 minutes to complete.

To begin, we have just a few general questions for you. Simply click on the “Continue” button at the bottom of the page to begin the survey.

***_______________________________________________________________________________***

***Demographic data : Age, education, Socio-economic status, location (provided from panel detail)***

**The first few questions are about your young children and what types of foods you prepare for them.**

**Screening Questions (All participants)**

S0. Please select the city you live within or live close to.

THAILAND (PN: Show drop down if from Thailand, TERMINATE anyone who answers other than 1)

1. Bangkok -🡪 CONTINUE
2. Nonthaburi
3. Nakhon Ratchasima
4. Chiang Mai
5. Other

INDONESIA (PN: Show drop down if from Indonesia, TERMINATE anyone who answers other than 1)

1. Jakarta -🡪 CONTINUE
2. Surabaya
3. Bandung
4. Bekasi
5. Other

MALAYSIA (PN: Show drop down if from Malaysia, TERMINATE anyone who answers other than 1)

1. Kuala Lumpur -🡪 CONTINUE
2. Seberang Perai
3. Subang Jaya
4. George Town
5. Other

PHILIPPINES ((PN: Show drop down if from Philippines, TERMINATE anyone who answers other than 1)

1. Manila🡪 CONTINUE
2. Cebu
3. Davao
4. Quezon
5. Other

VIETNAM ((PN: Show drop down if from Philippines, TERMINATE anyone who answers other than 1)

1. Hanoi 🡪 CONTINUE
2. Ho Chi Minh
3. Da Nang
4. Haiphong
5. Other

S1 (ASK ALL) Please specify your gender?

1. Male **🡪 TERMINATE**

2. Female

S1a. (ASK ALL) Do you have any children?

1. Yes
2. No -🡪 **TERMINATE**

S1b. (ASK ALL) How old is your youngest child (in months)?

1. 0- 5 months >> **TERMINATE**
2. 6 - 11 months >> **CONTINUE**
3. 12 -24 months >> **CONTINUE**
4. Older than 24 months >> **TERMINATE**

S2. (ASK ALL) Does your youngest child eat solid/semi-solid/soft foods?

1. Yes
2. No -🡪 **TERMINATE**

S3. (ASK ALL) Do you currently purchase packaged complementary foods (ADD HOVER TEXT OR ICON NEXT TO THIS AND INSERT TEXT FROM *You may refer know of them as ‘****baby foods’*** *or* ***‘toddler foods’****.  Specifically, these foods are products that are either packaged as ready-to-eat for the child in a jar, pouch, bag, box or other container, or prepared with the addition of water or milk or other liquids. Packaged complementary foods include* ***cereals*** *(like cerelac, or baby rice),* ***full meals*** *(such as pureed chicken and rice),* ***meal components*** *(for example vegetable purees, fruit purees),* ***desserts*** *(baby custards) and* ***snacks*** *(like biscuits, puffs, fruit snacks). Packaged complementary foods are specifically labelled and marketed for children up to the age of 3.”*) for your youngest child that is aged 6-24 months?

1. Yes - SKIP TO **S4**
2. No

S3a. (ASK IF S3=2; MULTI SELECT; RANDOMIZE) If no, what is the main reason you don’t purchase packaged complementary foods?

(*select all that apply*)

1. I don't like to buy processed foods
2. My child doesn’t like them
3. I don't know if they are good for my child
4. I think they are unhealthy
5. They are too expensive/Cannot afford to buy
6. They are not available in stores or markets near me/where I shop
7. Other, please specify _________ (INSERT TEXTBOX)

S4. (ASK ALL; MULTI SELECT) What type of foods do **you** (the primary caregiver) currently provide to this child?

*(Select all that apply)*

1. Home cooked food (meal specifically prepared for child)
2. Home cooked food (same dish/recipe as eaten by family)
3. Prepared food from outside of the home (Street food vendor, takeaway meal from restaurant/market)
4. Packaged complementary foods from the market/shop/grocery/pharmacy/online (SHOW IF S3=1)
5. Fresh fruits/vegetables
6. Other (please describe)_________ (INSERT TEXTBOX)

PROGRAMMING NOTES

- Participants who do not purchase packaged complementary foods (S3) now exit the survey TERMINATE IF S3=2
- Participants who answered 1/4 AT S1 AND yes (CODE 1) to S2 and S3 continue on to the next section

**The next set of questions are about the purchasing and use of packaged complementary foods. These questions are ONLY about packaged complementary foods, and not other types of foods that are suitable/marketed for children and adults. Please answer the question for your youngest child that is consuming packaged complementary foods.**

U1. (ASK ALL) **In the past month**, how often have you given your child packaged complementary foods?

1. Rarely (a few times in the past month or less)
2. Every few days
3. About once a day
4. At most feedings/meal
5. Every feeding/meal

U2. (ASK ALL; MUTLI SELECT, RANDOMIZE ORDER)

What are your reasons for purchasing the packaged complementary food(s)

(select all that apply)

1. Child asked for it/demanded it
2. Given as a treat/gift
3. Given to calm child down/ stop them from crying
4. Given to keep child busy or entertained/ distract child
5. Child likes eating it/the taste
6. Can be fed to child easily/fed without assistance because of its taste/shape/texture
7. Easy to prepare/ready-to-eat
8. Readily available/close by
9. I/other people think it’s nutritious/good for child's health/ development
10. I/other people think it’s safe/clean
11. Package/ advertisements say it’s nutritious/good for child’s health/ development
12. Someone else was eating it
13. It is affordable/inexpensive
14. To diversify diet/introduce a new food/taste
15. Other (specify)__________ (ANCHOR; INSERT TEXTBOX)
16. Don’t Know ________ (ANCHOR; EXCLUSIVE)

U2a. (ASK ALL; SINGLE ANSWER) What is your **main** reason for purchasing complementary food(s)? Please select one.

(PN:Pipe in answers from U2, including those in 15. “Other, specify”)

1. Child asked for it/demanded it
2. Given as a treat/gift
3. Given to calm child down/ stop them from crying
4. Given to keep child busy or entertained/ distract child
5. Child likes eating it/the taste
6. Can be fed to child easily/fed without assistance because of its taste/shape/texture
7. Easy to prepare/ready-to-eat
8. Readily available/close by
9. I/other people think it’s nutritious/good for child's health/ development
10. I/other people think it’s safe/clean
11. Package/ advertisements say it’s nutritious/good for child’s health/ development
12. Someone else was eating it
13. It is affordable/inexpensive
14. To diversify diet/introduce a new food/taste

U3. (ASK ALL) How frequently do you buy packaged complementary foods?

1. Every couple of months
2. Monthly (about once a month)
3. Multiple times per month
4. Weekly (about once a week)
5. Multiple times a week

U4. (ASK ALL) Where do you **most frequently** purchase packaged complementary foods from? (select the **one** place you buy from most frequently)

1. Supermarket (in store)
2. Local market
3. Mini mart
4. Pharmacy
5. Baby store
6. Online supermarket/grocery
7. Other Online store (please name)……… (INSERT TEXTBOX)
8. Other(please name)______ (ANCHOR LAST; INSERT TEXTBOX)

PN: provide examples for specific countries:

| **Country** | **Type of Store** | **Example** |
| --- | --- | --- |
| Vietnam | Supermarket (in store) | Big C |
|  | Local market |  |
|  | Mini mart |  |
|  | Pharmacy |  |
|  | Baby store | ConCung |
|  | Online supermarket/grocery |  |
| Thailand | Supermarket (in store) | Big C, TOPS |
|  | Local market |  |
|  | Mini mart | 7/11 |
|  | Pharmacy | Watson, Save Drug |
|  | Baby store | Bonny Kid, ABC the Baby |
|  | Online supermarket/grocery |  |
| Indonesia | Supermarket (in store) | Transmart Carrefour |
|  | Local market |  |
|  | Mini mart |  |
|  | Pharmacy |  |
|  | Baby store | Chubby Baby Shop. |
|  | Online supermarket/grocery |  |
| Malaysia | Supermarket (in store) | Village Grocer , Mercato |
|  | Local market |  |
|  | Mini mart |  |
|  | Pharmacy | Big pharmacy, AA Pharmacy |
|  | Baby store | Babyland, Happikiddo |
|  | Online supermarket/grocery |  |
| Philippines | Supermarket (in store) | Robinsons Supermarket, |
|  | Local market |  |
|  | Mini mart |  |
|  | Pharmacy |  |
|  | Baby store | Babyzone |
|  | Online supermarket/grocery |  |

U5. (ASK ALL; MULTI SELECT; RANDOMISE ORDER)

What type of packaged complementary foods do you purchase for your child aged 6-24 months (under 2)?

1. Cereals and porridges

2. Purees (vegetable or fruits only)

3. Foods/Meals that contain meats (eggs, fish, beef, chicken, pork, lamb)

4. Snack foods

5. Dairy products (excluding beverages) - baby cheeses, yoghurts

6. Other (please describe/name product)__________ (ANCHOR; INSERT TEXTBOX)

U5a (ASK IF U5=2; MULTI SELECT)

You mentioned that you purchase **Purees** for your child. What type of purees do you purchase for your child aged 6-24 months (under 2)?

Select all that apply

- - 1. Fruit
    2. Veg
    3. Mix of fruit and veg

U5b (ASK IF U5=4; MULTI SELECT)

You mentioned that you purchase **Snack Foods** for your child. What type of Snack food do you purchase for your child aged 6-24 months (under 2)? Select all that apply

- - 1. Savoury snacks (puffs, crackers, vegetable chips)
    2. Fruit snacks (dried fruit)
    3. Sweet snacks (youghurt bites, fruit gummies, sweet biscuits)

U6. (ASK ALL; MULTI SELECT)

At what times do you **usually** give a packaged complementary food to your youngest child? (select all that apply)

1. As a snack/between meals
2. As a full meal at home
3. When out of the house
4. To take to daycare/childcare
5. Other (please specify)_____ (INSERT TEXTBOX)

U7. (ASK ALL; MULTI SELECT; RANDOMIZE ORDER)

What **factors** influence your decision to purchase a packaged complementary food?

(select all that apply)

1. The mix of ingredients
2. Foods I would not usually prepare for the child at home
3. Health information
4. Low/no sugar
5. Low/no added salt
6. Nutritional value
7. Price
8. Convenience
9. Quality of ingredients
10. Other (please state)_______ (ANCHOR; INSERT TEXTBOX)

U9. (ASK ALL; MULTI SELECT; RANDOMIZE ORDER)

What **concerns** do you have when buying packaged complementary foods for your child? (Select all that apply)

1. The sugar content
2. The salt content
3. The nutritional value
4. The quality of ingredients
5. The product might be unsafe/tampered with
6. Price
7. Other (please state)____ (ANCHOR; INSERT TEXTBOX)

**Labelling of Packaged Complementary Foods - these questions are about the types of labels and information included on packaged complementary foods. Labels can include different types of nutrition information – including details on calories, salt and sugars, and claims about the products themselves – for example a product may have a label that says “good source of protein” or “good for growing bones”**

**[INSERT IMAGES PER COUNTRY, DISPLAY IMAGES FOR QUESTIONS L1-L5]**

[L1 Images: Only nutrition]

Indonesia:

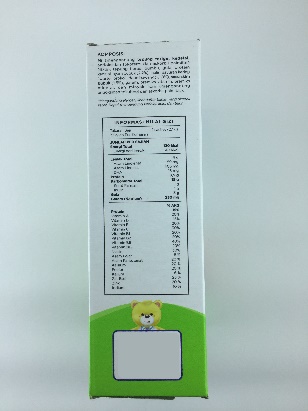


Malaysia

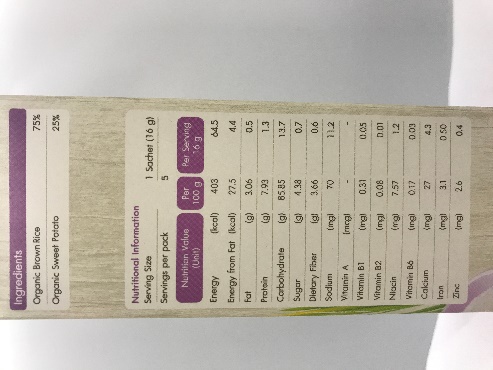


Philippines

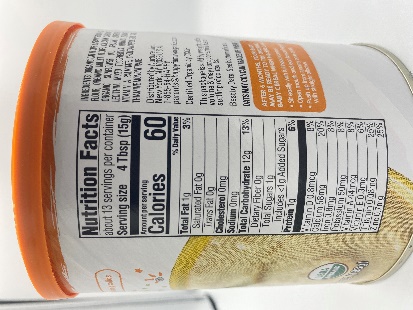


Thailand

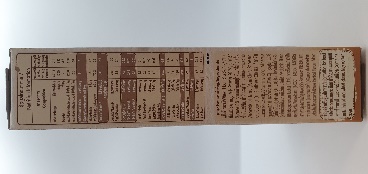


Vietnam

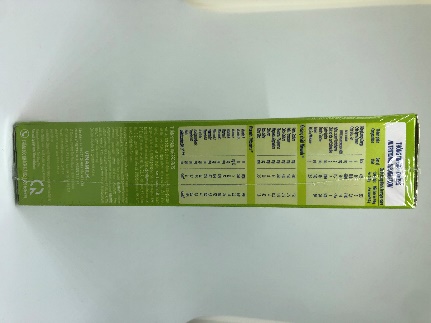


L1. (ASK ALL) When you buy a packaged complementary food for the first time how often do you read the **nutritional information** (this is usually provided in a box, and includes information on the fat, salt, sugar, protein and carbohydrate composition )? See example above.

1. Often
2. Sometimes
3. Rarely
4. Never

[L2 &L3 Images are claims only]

Indonesia:

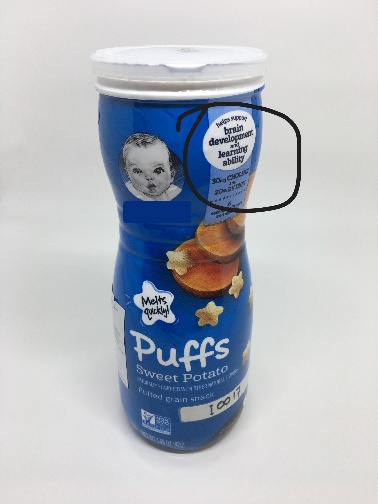


Malaysia

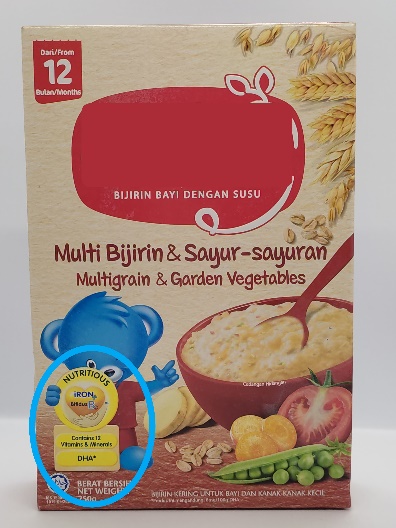


Philippines

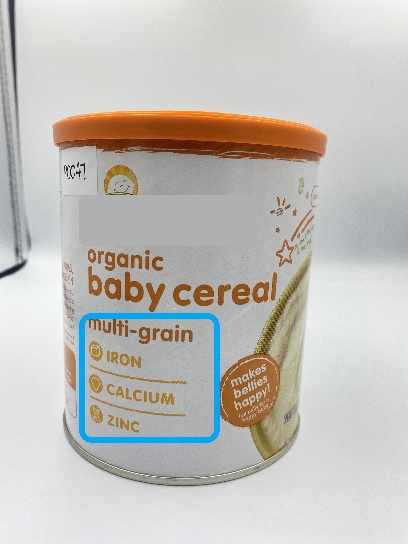


Thailand

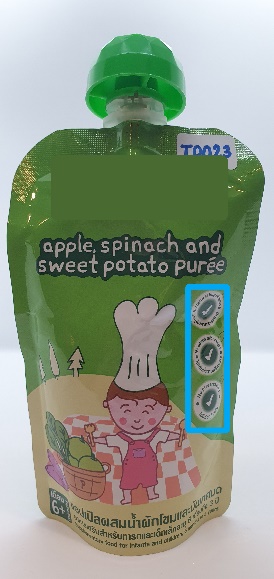


Vietnam

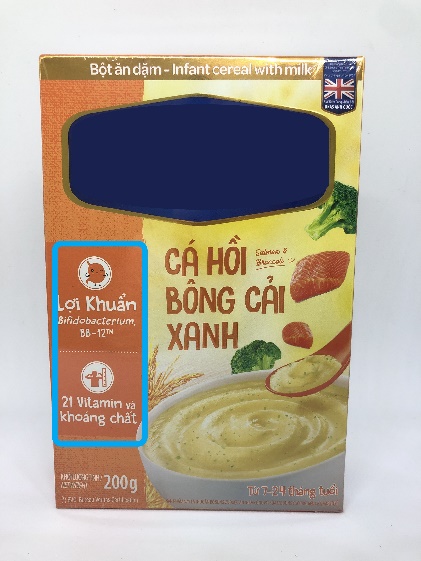


L2. (ASK ALL) How often do you **refer to** the label claims about **vitamin content** (e.g high in vitamin c, good source of vitamin a – see example above) on packaged complementary food for your child,

1. Often
2. Sometimes
3. Rarely
4. Never

[SAME IMAGES AS L2]

L3. (ASK ALL) When a packaged complementary food for your child has a claim about **vitamin** **content claim** (e.g. high in vitamin c, good source of vitamin A - see example above ) how often do you trust the claim (i.e. that the product actually is high in vitamin c)?

1. Often
2. Sometimes
3. Rarely
4. Never

[L4 IMAGES: The same as before + the nutrition images]

Indonesia:


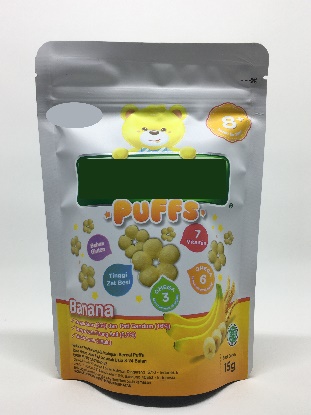

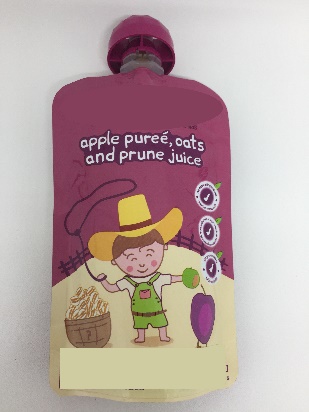

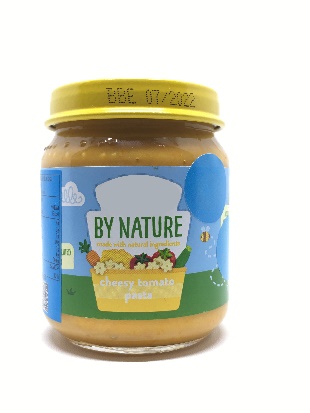

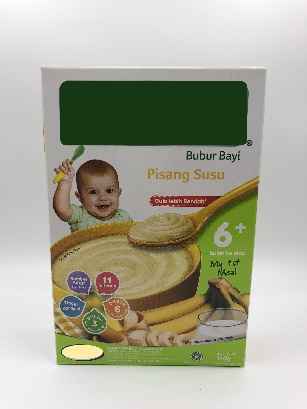

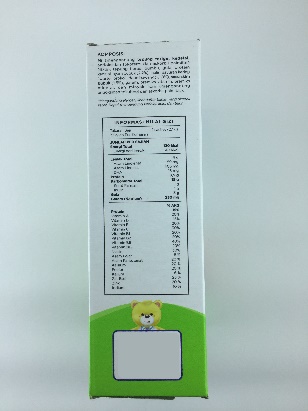


Malyasia


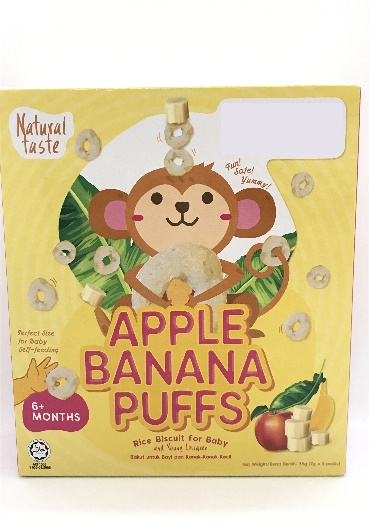

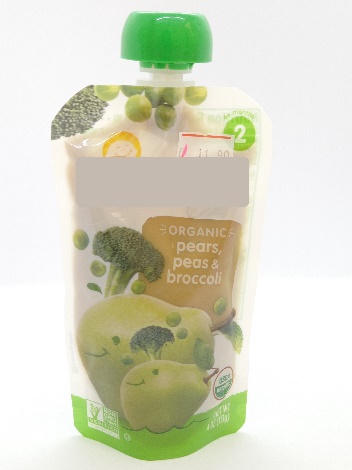

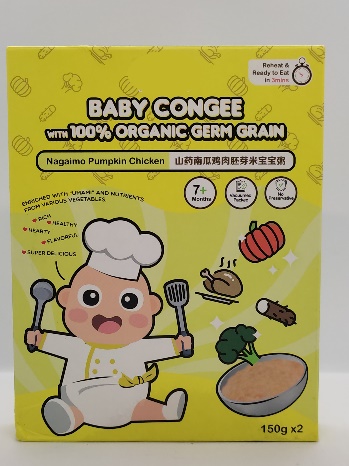

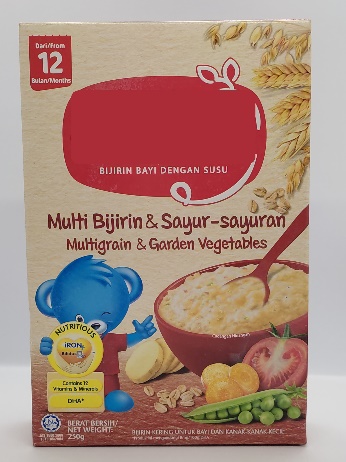


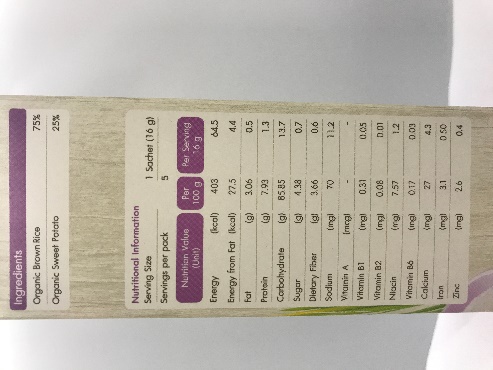


Philippines


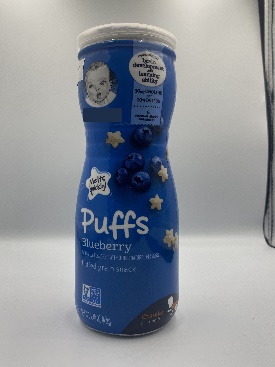

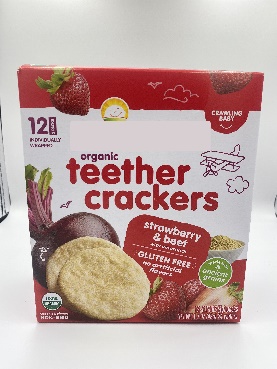

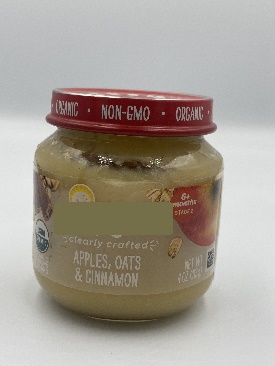

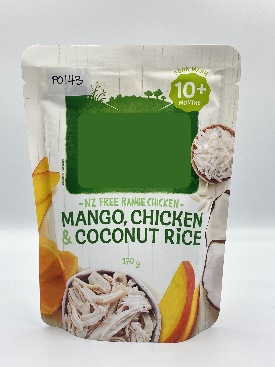

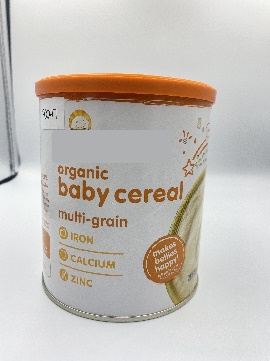


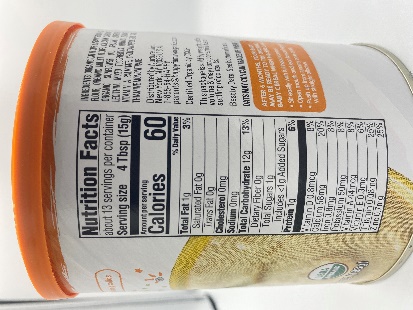


Thailand


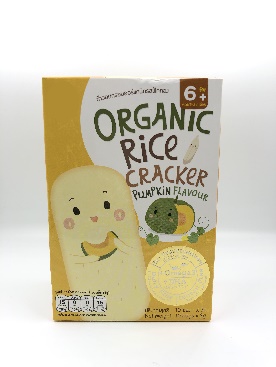

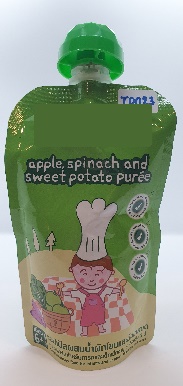

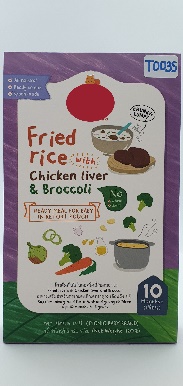

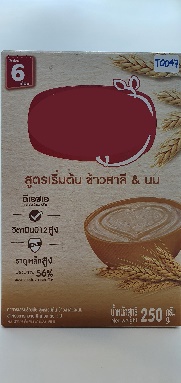

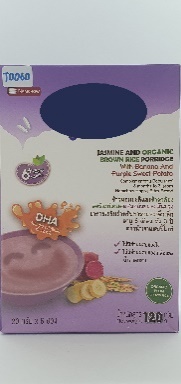


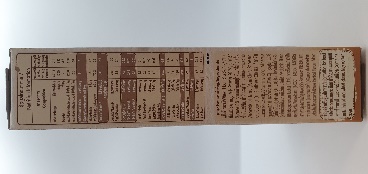


Vietnam


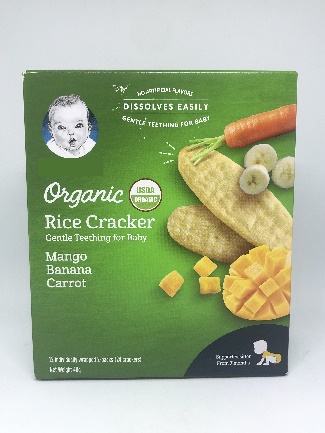

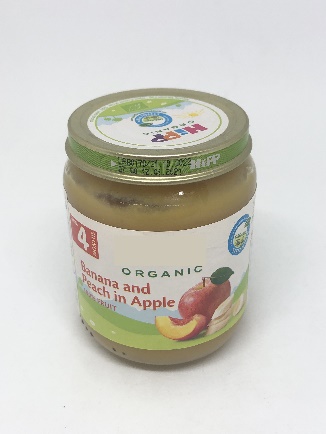

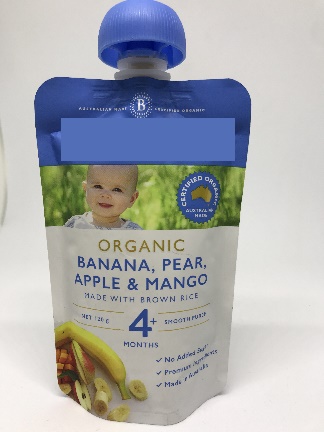

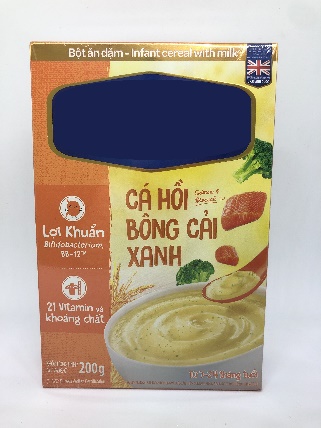

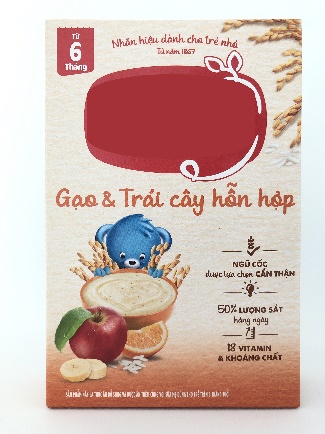

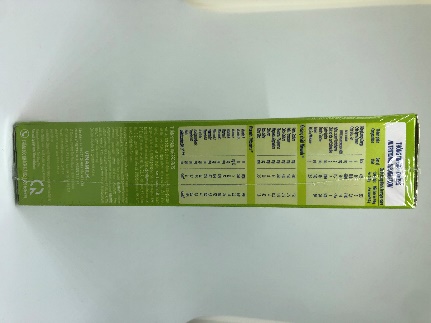


L4. (ASK ALL; MULTI SELECT; RANDOMIZE ORDER)

What label information or part of the package do you look at when you are looking to buy a packaged complementary food? (select all that apply)

1. List of ingredients
2. Nutrition information
3. Statements about health and nutrition benefits
4. Images/design
5. Food labelling on the front of the packet
6. Name/brand
7. Other (please describe)_____ (ANCHOR; INSERT TEXTBOX)

L5. (ASK ALL; MULTI SELECT; RANDOMIZE ORDER)

Which of the following terms, if you saw them on the front label of a packaged complementary food would make you more likely to purchase that product for your child compared to a similar product without those terms?^[[4]](#footnote-5)^ (select all that apply)

1. Low sugar
2. Low sodium/salt
3. No artificial ingredients
4. No artificial colours
5. No added sugar
6. Natural
7. Organic
8. Healthy
9. Fortified
10. None of these would make me more likely to purchase that product……..(ANCHOR; EXCLUSIVE) **SKIP TO S1**

L6. . (ASK if L5 =/= 10, SINGLE SELECT)

Which of the terms in the previous question (PN : Pipe the responses from L5) is most important to you? (please select one)

1. Low sugar
2. Low sodium/salt
3. No artificial ingredients
4. No artificial colours
5. No added sugar
6. Natural
7. Organic
8. Healthy
9. Fortified

**­­­­­­­­**

**Source of Information**

**The final question is about where you go for advice and information on feeing your children.**

S1. (ASK ALL; MULTI SELECT; RANDOMIZE ORDER)

Where do you go for reliable information on feeding your child? (select all that apply)

1. Doctor/health care professionals
2. Mother/mother-in-law
3. Other family member
4. Friends
5. Online – web search (please name specific website)____(ANCHOR SECOND LAST) (INSERT TEXTBOX)
6. Online - parent group
7. Social media
8. Parent/mothers group
9. Other (please specify)____ ___ (ANCHOR; INSERT TEXTBOX)

**Demographic Questions**

D1. Please enter your age:

- - - 1. INSERT NUMERIC TEXTBOX
      2. Prefer not to say

**D1. [HIDDEN VARIABLE – AGE BRACKETS ]**

| AGE BRACEKTS | Hidden Variable |
| --- | --- |
| 18-24 | 1 |
| 25-35 | 2 |
| 36-45 | 3 |
| 46-50 | 4 |
| 51-55 | 5 |
| 56-60 | 6 |
| 61-65 | 7 |
| 66 and more | 8 |

D2. How many family members live in your household?

*The total family member who live together and sharing food in the same kitchen. Including children in any range of age, not including workers who works in the household (maid/chauffeur etc) and guest.*

- - - 1. INSERT NUMERIC TEXTBOX
      2. Prefer not to say

D3. What is your highest level of education?

1. Elementary School or lower
2. Junior High School
3. High School
4. Vocational School
5. College / University for Bachelor’s degree or equivalent
6. Master’s Degree
7. Doctoral Graduate

98. Prefer not to say

**PN: SEC questions to be asked in router**

**SEC Calculation per country**

D4. What is your monthly household income?

**Philippines**

1. Less than 5,000 Peso
2. 5000 - 9,999 Peso
3. 10,000 - 19,999 Peso
4. 20,000 - 39,999 Peso
5. 40,000 - 99,999 Peso
6. 100,000 - 199,999 Peso
7. 200,000 Peso or more

**Indonesia**

1. Rp 2,500,000 or less
2. Rp 2,500,001 - 5,000,000
3. Rp 5,000,001 - 10,000,000
4. Rp 10,000,001 - 15,000,000
5. Rp 15,000,001 - 20,000,000
6. Rp 20,000,001 - 25,000,000
7. Rp 25,000,001 or more

**Vietnam**

How much was your average **monthly** household earnings (before taxes) in 2021? Please include the before-tax income of all earning members in the household living with you.

|  |  | Code |
| --- | --- | --- |
| 1 | 1,500,000 - 2,999,999 VND | HIB E |
| 2 | 3,000,000 - 4,499,999 VND | HIB D |
| 3 | 4,500,000 - 7,499,999 VND | HIB C |
| 4 | 7,500,000 - 14,999,999 VND | HIB B |
| 5 | 15,000,000 VND or more | HIB A |

**Indonesia**

SEC1. Pipe in HH number from D2

SEC2. How much is your average household expenditure in a month? (Coming from purchases, own production and giving).

**For food expenditures including :**

Rice, corn, wheat, rice flour, corn flour, cassava, sweet potato, potato, cassava, taro, sago,

Fish / shrimp / squid / mussels fresh / salted / preserved, meat, eggs, milk, vegetables, nuts, fruits,

Oil, beverage ingredients (sugar, tea, coffee, etc.), seasonings, instant noodles, crackers, bread, biscuits, porridge, meatballs, soft drinks, ice syrup, mineral water, alcohol, tobacco sticks, hand-rolled cigarettes, etc.

**For non-food expenditures including:**

Lease, contract, estimates of the rent per month,

Electricity bills, telephone, gas, kerosene,

Account home phone, toll-HP, public telephones, kiosks,

Bath soap / shampoo, cosmetics, hair care / face, tissue,

Cost of Education, Transportation, transportation, gasoline, diesel fuel, lubricating oils,

Levies / charges, insurance paid per month

| **Range** | **Code** | **Score / HH Size** | | | | | | | | | | | | | | | | | | |  |
| --- | --- | --- | --- | --- | --- | --- | --- | --- | --- | --- | --- | --- | --- | --- | --- | --- | --- | --- | --- | --- | --- |
|  |  | 1 | 2 | 3 | 4 | 5 | 6 | 7 | 8 | 9 | 10 | 11 | 12 | 13 | 14 | 15 | 16 | 17 | 18 | 19 | 20 |
| Up to Rp 300.000 | 01 | 1 | 1 | 1 | 1 | 1 | 1 | 1 | 1 | 1 | 1 | 1 | 1 | 1 | 1 | 1 | 1 | 1 | 1 | 1 | 1 |
| Rp 300.001 - Rp 400.000 | 02 | 2 | 1 | 1 | 1 | 1 | 1 | 1 | 1 | 1 | 1 | 1 | 1 | 1 | 1 | 1 | 1 | 1 | 1 | 1 | 1 |
| Rp 400.001 - Rp 500.000 | 03 | 4 | 1 | 1 | 1 | 1 | 1 | 1 | 1 | 1 | 1 | 1 | 1 | 1 | 1 | 1 | 1 | 1 | 1 | 1 | 1 |
| Rp 500.001 - Rp 600.000 | 04 | 5 | 2 | 1 | 1 | 1 | 1 | 1 | 1 | 1 | 1 | 1 | 1 | 1 | 1 | 1 | 1 | 1 | 1 | 1 | 1 |
| Rp 600.001 - Rp 700.000 | 05 | 6 | 2 | 1 | 1 | 1 | 1 | 1 | 1 | 1 | 1 | 1 | 1 | 1 | 1 | 1 | 1 | 1 | 1 | 1 | 1 |
| Rp 700,001 - 750,000 | 06 | 6 | 3 | 1 | 1 | 1 | 1 | 1 | 1 | 1 | 1 | 1 | 1 | 1 | 1 | 1 | 1 | 1 | 1 | 1 | 1 |
| Rp 750,001 - 800,000 | 07 | 6 | 3 | 2 | 1 | 1 | 1 | 1 | 1 | 1 | 1 | 1 | 1 | 1 | 1 | 1 | 1 | 1 | 1 | 1 | 1 |
| Rp 800,001 - 900,000 | 08 | 7 | 4 | 2 | 1 | 1 | 1 | 1 | 1 | 1 | 1 | 1 | 1 | 1 | 1 | 1 | 1 | 1 | 1 | 1 | 1 |
| Rp 900,001 - 1,000,000 | 09 | 7 | 4 | 2 | 1 | 1 | 1 | 1 | 1 | 1 | 1 | 1 | 1 | 1 | 1 | 1 | 1 | 1 | 1 | 1 | 1 |
| Rp 1,000,001 - 1,250,000 | 10 | 7 | 5 | 2 | 2 | 1 | 1 | 1 | 1 | 1 | 1 | 1 | 1 | 1 | 1 | 1 | 1 | 1 | 1 | 1 | 1 |
| Rp 1.250,001 - 1,500,000 | 11 | 7 | 6 | 4 | 2 | 2 | 1 | 1 | 1 | 1 | 1 | 1 | 1 | 1 | 1 | 1 | 1 | 1 | 1 | 1 | 1 |
| Rp 1,500,001 - 1,750,000 | 12 | 8 | 6 | 5 | 3 | 2 | 2 | 1 | 1 | 1 | 1 | 1 | 1 | 1 | 1 | 1 | 1 | 1 | 1 | 1 | 1 |
| Rp 1.750,001 - 2,000,000 | 13 | 8 | 7 | 6 | 4 | 3 | 2 | 2 | 1 | 1 | 1 | 1 | 1 | 1 | 1 | 1 | 1 | 1 | 1 | 1 | 1 |
| Rp 2,000,001 - 2,250,000 | 14 | 8 | 7 | 6 | 5 | 4 | 2 | 2 | 2 | 1 | 1 | 1 | 1 | 1 | 1 | 1 | 1 | 1 | 1 | 1 | 1 |
| Rp 2,250,001 - 2,500,000 | 15 | 8 | 7 | 6 | 6 | 4 | 3 | 2 | 2 | 2 | 1 | 1 | 1 | 1 | 1 | 1 | 1 | 1 | 1 | 1 | 1 |
| Rp 2,500,001 - 2,750,000 | 16 | 8 | 7 | 7 | 6 | 5 | 4 | 3 | 2 | 2 | 2 | 1 | 1 | 1 | 1 | 1 | 1 | 1 | 1 | 1 | 1 |
| Rp 2,750,001 - 3,000,000 | 17 | 8 | 8 | 7 | 6 | 6 | 4 | 3 | 3 | 2 | 2 | 2 | 1 | 1 | 1 | 1 | 1 | 1 | 1 | 1 | 1 |
| Rp 3,000,001 - 3,250,000 | 18 | 9 | 8 | 7 | 6 | 6 | 5 | 4 | 3 | 2 | 2 | 2 | 2 | 1 | 1 | 1 | 1 | 1 | 1 | 1 | 1 |
| Rp 3,250,001 - 3,500,000 | 19 | 9 | 8 | 7 | 7 | 6 | 6 | 4 | 4 | 3 | 2 | 2 | 2 | 2 | 1 | 1 | 1 | 1 | 1 | 1 | 1 |
| Rp 3.500.001 - Rp 4.000.000 | 20 | 9 | 8 | 7 | 7 | 6 | 6 | 5 | 4 | 3 | 3 | 2 | 2 | 2 | 2 | 1 | 1 | 1 | 1 | 1 | 1 |
| Rp 4.000.001 - Rp 4.500.000 | 21 | 9 | 8 | 7 | 7 | 7 | 6 | 6 | 5 | 4 | 4 | 3 | 2 | 2 | 2 | 2 | 2 | 1 | 1 | 1 | 1 |
| Rp 4.500.001 - Rp 5.000.000 | 22 | 9 | 8 | 8 | 7 | 7 | 6 | 6 | 6 | 5 | 4 | 4 | 3 | 3 | 2 | 2 | 2 | 2 | 2 | 1 | 1 |
| Rp 5.000.001 - Rp 6.000.000 | 23 | 9 | 8 | 8 | 7 | 7 | 7 | 6 | 6 | 6 | 5 | 4 | 4 | 3 | 3 | 2 | 2 | 2 | 2 | 2 | 2 |
| Rp 6.000.001 - Rp 7.000.000 | 24 | 9 | 9 | 8 | 8 | 7 | 7 | 7 | 6 | 6 | 6 | 6 | 5 | 4 | 4 | 4 | 3 | 3 | 2 | 2 | 2 |
| Rp 7.000.001 - Rp 8.000.000 | 25 | 9 | 9 | 8 | 8 | 8 | 7 | 7 | 7 | 7 | 6 | 6 | 6 | 6 | 5 | 4 | 4 | 4 | 3 | 3 | 3 |
| Rp 8.000.001 - Rp 9.000.000 | 26 | 9 | 9 | 8 | 8 | 8 | 7 | 7 | 7 | 7 | 7 | 6 | 6 | 6 | 6 | 6 | 5 | 5 | 4 | 4 | 4 |
| Rp 9.000.001 - Rp 10.000.000 | 27 | 9 | 9 | 9 | 8 | 8 | 8 | 7 | 7 | 7 | 7 | 7 | 6 | 6 | 6 | 6 | 6 | 6 | 5 | 5 | 4 |
| Rp 10,000,001 - 12,500,000 | 28 | 9 | 9 | 9 | 8 | 8 | 8 | 8 | 7 | 7 | 7 | 7 | 7 | 6 | 6 | 6 | 6 | 6 | 6 | 6 | 5 |
| Rp 12,500,001 - 15,000,000 | 29 | 9 | 9 | 9 | 9 | 8 | 8 | 8 | 8 | 8 | 7 | 7 | 7 | 7 | 7 | 7 | 7 | 6 | 6 | 6 | 6 |
| More than Rp 15,000,000 | 30 | 9 | 9 | 9 | 9 | 9 | 8 | 8 | 8 | 8 | 8 | 8 | 7 | 7 | 7 | 7 | 7 | 7 | 7 | 7 | 6 |

SEC3.

| What kind of fuel is most commonly used in the household for daily cooking needs? [SA] | Code | Score |
| --- | --- | --- |
| Electricity | 01 | 5 |
| LPG Gas – 12 KG/Gas 5 KG/Gas 7 KG | 02 | 4 |
| LPG Gas - 3 KG | 03 | 2 |
| Natural Gas (in KG) | 04 | 4 |
| Kerosene | 05 | 2 |
| Wood | 06 | 1 |
| Charcoal | 07 | 1 |
| Briquet | 08 | 1 |
| Others | 09 | 1 |
| Not cooking | 10 |  |
| If HH expenditure < 2,5mil per month (SEC2 code 1-15) |  | 2 |
| If HH expenditure 2,5mil – 5mil per month (SEC 2 code 16-22) |  | 3 |
| If HH expenditure > 5mil per month (SEC 2 code 23-30) |  | 4 |

| SEC 4 | What kind of sources of drinking water are most commonly used in the household? [SA][SA] | Code | Score |
| --- | --- | --- | --- |
|  | Branded bottled water | 01 | 4 |
|  | Refill drinking water | 02 | 3 |
|  | Metered tap water | 03 | 2 |
|  | Retailed tap water | 04 | 1 |
|  | Drilled/pumped well | 05 | 1 |
|  | Sheltered well | 06 | 0 |
|  | Not sheltered well | 07 | 0 |
|  | Sheltered spring | 08 | 0 |
|  | Not sheltered spring | 09 | 0 |
|  | River water | 10 | 0 |
|  | Rain water | 11 | 0 |
|  | Others | 12 | 0 |

PN: Calculate all score

| SCORE SEC 2 | SCORE SEC 3 | SCORE SEC 4 | **Total Score** |
| --- | --- | --- | --- |
|  |  |  |  |

| SEC 5 | Code the SEC group based on table range below | |
| --- | --- | --- |
|  |  | Code |
|  | 15 – 18 (Upper 1) | 1 |
|  | 13 – 14 (Upper 2) | 2 |
|  | 11 – 12 (Middle 1) | 3 |
|  | 8 – 10 (Middle 2) | 4 |
|  | 5 – 7 (Lower 1) | 5 |
|  | 2 – 4 (Lower 2) | 6 |

**Philippines**

**INSTRUCTION:**Now you will be asked a few questions about you and your household. Rest assured that these will only be used for this study and it will be maintained in strict confidentiality.

**FACTUALS TO ESTABLISH SEC:**

| **SEC08. Status of Housing Unit [SA]**  Please choose below which one best describes the status of your housing unit | | **SEC11. Vehicle Ownership**  Do you have any cars? If yes, is the car amoritized or fully-owned? | | **SEC14. Fuel for Cooking [MA]**  Which of the items below do you use for cooking? | |
| --- | --- | --- | --- | --- | --- |
| Fully-owned | 5 | Cars/UV/SUV **[SA]** |  | Electricity | 1 |
| Amortized | 4 | Fully-owned | 2 | LPG | 1 |
| Rented | 2 | Amortized | 1 | Others | - |
| House owned but lot not owned | 1 | None | - | **SEC15. Education of HH/CIE [SA]**  Please select which of these is the highest educational attainment of your household head/chief income earner (the one who mainly provides for the household). | |
| Not owned (rent-free) |  | SEC11a. What is the age of your latest car/automobile?  Least age of vehicle: ______ |  |  |  |
| **SEC09. Type of Housing Unit [SA]**  Which type of housing unit does your household most likely fall under? | | If age <= 5yrs… new vehicle | 1 | No schooling | - |
| Multi-storey | 5 | **SEC12. Household Durables [MA]**  Now, please select below all of the items that you have in your household. | | Elementary or less | 1 |
| Bungalow (detached) |  | Air-Conditioner | 1 | Some high school | 2 |
| Duplex/ Triplex |  | Digital Camera | 1 | High school graduate | 3 |
| Townhouse |  | Electric Fan | 1 | Some college/ vocational |  |
| Condominium |  | Flat Iron | 1 | College graduate of SUCs | 4 |
| Apartment house | 4 | Internet Subscription | 1 | Some masteral |  |
| Row house (e.g., pabahay) | 3 | Microwave Oven | 1 | College graduate of exclusive UCs | 5 |
| Single-storey (attached) |  | Refrigerator | 1 | Masteral/ doctoral graduate |  |
| Room-only/ Studio-type | 2 | Washing Machine | 1 | **SEC16. Occupation of HH/CIE [SA]**  Which of the following best describes the current occupation of your household head/chief income earner (the one who mainly provides for the household)? | |
| Nipa/ Makeshift house | 1 | **SEC13. Monthly Electricity Bill [SA]**  Please select in which range your monthly electricity bill most likely falls under | |  |  |
| **SEC10. Toilet Facility**  Please input below the number of toilet facilities you have in your house. Please select the type of facilities you have in your house. Please select all that applies. | | Average spent: __________ |  | Unskilled  (baker attendant, factory worker, fisherman (small time), hospital attendant, farm helper, houseboy, laborer, laundry woman, time keeper, vendor, etc.) | 1 |
| # of Toilet Facility: ______ |  | None/ Free | - | Blue-collar  (retiree, Phil veteran/soldier, baker, carpenter/furniture maker, electrician/mechanic, fireman/policeman, professional midwife, driver/PUJ driver, office employee, sales clerk, etc) | 2 |
| If n=0… no own toilet | -1 | P1 – P750 | 1 | White-collar  (customs inspector, social worker, salesman, labor foremen, small land owner, small-time real estate agent/broker, any commodity dealer, etc.) | 3 |
| If n=1… with flush (in/out) | 1 | P751 – P1,500 | 2 | Middle management  (junior architect/engineer, business adviser/consultant, resident physician, professor, news reporter, journalist of newspaper, mid-level manager of big companies, real estate agent or broker, etc.) | 4 |
| If n=1… de buhos (in) | - | P1,501 - P3,000 | 3 | Have small business  (bakery, sari-sari store, transportation, jeep operator, few tricycles, etc.) |  |
| If n=1… de buhos (out) | -1 | P3,001 – P6,000 | 4 | Professional/ Senior management  Senior architect/engineer, lawyer, dentist, specialist physician, pilot, big-time radio/tv announcer, business executive (senior/top management), government officials, etc.) | 5 |
| If n>1… with flush (in) | 1 | P6,001 ++ | 5 | Have medium to large business  (grocery, restaurant, supermarket, large restaurant, hacienda owner, real estate proprietor/developer, etc.) |  |

| **SEC17/SEC18 Scoring System** | | | |
| --- | --- | --- | --- |
| **Class** | **Min** | **-** | **Max** |
| AB | 34 | - | 40 |
| C1 | 27 | - | 33 |
| C2 | 20 | - | 26 |
| D | 13 | - | 19 |
| *D1* | *17* | - | *19* |
| *D2* | *15* | - | *16* |
| *D3* | *13* | - | *14* |
| E | 3 | - | 12 |
| *E1* | *10* | - | *12* |
| *E2* | *3* | - | *9* |

**Thailand**

AUTOCODE Q14=1 (SINCE RESPONDENT IS FROM BANGKOK)

| Q14  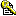 | AUTO CODE  **Auto code 1 if [Q12 = 1 AND Q13 = OR(1,2)]**  **Auto code 2 if [Q12 = OR(2,3,4,5) AND Q13 = 1]**  **Auto code 3 if [Q12 = OR(2,3,4,5) AND Q13 = 2]** | รหัส / โค้ด  (573) | เส้นทาง |
| --- | --- | --- | --- |
|  | GBKK | 1 |  |
|  | UPC Urban | 2 |  |
|  | UPC Rural | 3 |  |

| **Q15** | **SHOW CARD**  Please tell me which one in the card best describes your **average monthly household income**? Please include salary, bonus, stock profit and etc. [SA]  **แสดงการ์ด**  จากการ์ดนี้ กรุณาบอกหน่อยนะคะว่า **รายได้ครอบครัวเฉลี่ยต่อเดือน** ของคุณอยู่ในช่วงใด กรุณารวมเงินเดือน เงินโบนัส และ กำไรจากหุ้น และเงินผลประโยชน์อื่นๆ ด้วยนะคะ [คำตอบเดียว] | Code  (622) | Route |
| --- | --- | --- | --- |
|  | Less than 10,000 Baht  น้อยกว่า 10,000 บาท | 01 |  |
|  | 10,000-12,499 Baht  10,000-12,499 บาท | 02 |  |
|  | 12,500-14,999 Baht  12,500-14,999 บาท | 03 |  |
|  | 15,000-17,499 Baht  15,000-17,499 บาท | 04 |  |
|  | 17,500-19,999 Baht  17,500-19,999 บาท | 05 |  |
|  | 20,000-22,499 Baht  20,000-22,499 บาท | 06 |  |
|  | 22,500-24,999 Baht  22,500-24,999 บาท | 07 |  |
|  | 25,000-29,999 Baht  25,000-29,999 บาท | 08 |  |
|  | 30,000-34,999 Baht  30,000-34,999 บาท | 09 |  |
|  | 35,000-39,999 Baht  35,000-39,999 บาท | 10 |  |
|  | 40,000-44,999 Baht  40,000-44,999 บาท | 11 |  |
|  | 45,000-49,999 Baht  45,000-49,999 บาท | 12 |  |
|  | 50,000-54,999 Baht  50,000-54,999 บาท | 13 |  |
|  | 55,000-59,999 Baht  55,000-59,999 บาท | 14 |  |
|  | 60,000-64,999 Baht  60,000-64,999 บาท | 15 |  |
|  | 65,000-69,999 Baht  65,000-69,999 บาท | 16 |  |
|  | 70,000 - 79,999 Baht  70,000 -79,999 บาท | 17 |  |
|  | 80,000 – 89,999 Baht  80,000 –89,999 บาท | 18 |  |
|  | 90,000 – 99,999 Baht  90,000 –99,999 บาท | 19 |  |
|  | 100,000 Baht or above  100,000 บาท หรือ มากกว่า | 20 |  |

| **Q16** | **Programmer:**  **Auto code 1 if [Q14 = 1 AND Q15 = OR(15-20)] *OR* [Q14 = 2 AND Q15 = OR(13-20)] *OR* [Q14 = 3 AND Q15 = OR(11-20)]**  **Auto code 2 if [Q14 = 1 AND Q15 = OR(13,14)] *OR* [Q14 = 2 AND Q15 = OR(11,12)] *OR* [Q14 = 3 AND Q15 = OR(9,10)]**  **Auto code 3 if [Q14 = 1 AND Q15 = OR(9,10,11,12)] *OR* [Q14 = 2 AND Q15 = OR(6,7,8,9,10)] *OR* [Q14 = 3 AND Q15 = OR(4,5,6,7,8)]**  **Auto code 4 if [Q14 = 1 AND Q15 = OR(6,7,8)] *OR* [Q14 = 2 AND Q15 = OR(4,5)] *OR* [Q14 = 3 AND Q15 = OR(2,3)]**  **Auto code 5 if [Q14 = 1 AND Q15 = OR(1,2,3,4,5)] *OR* [Q14 = 2 AND Q15 = OR(1,2,3)] *OR* [Q14 = 3 AND Q15 = 1]**  **Interviewer** : Record code SES (check **Q15**) and check quota [SA]  **พนักงานสัมภาษณ์**: บันทึกรายได้ครอบครัว (เช็คข้อ **Q15**) และเช็คโควต้า [คำตอบเดียว] | Code  (632) | Route |
| --- | --- | --- | --- |
|  | A (GBKK 60,000 Baht or more, Urban 50,000 Baht or more, Rural 40,000 Baht or more)  A (กทม. 60,000 บาท หรือมากกว่า, Urban 50,000 บาท หรือมากกว่า, Rural 40,000 บาทหรือมากกว่า) | 1 |  |
|  | B (GBKK 50,000-59,999 Baht, Urban 40,000-49,999 Baht, Rural 30,000-39,999 Baht)  B (กทม. 50,000-59,999 บาท , Urban 40,000-49,999 บาท, Rural 30,000-39,999 บาท) | 2 |  |
|  | C (GBKK 30,000-49,999 Baht, Urban 20,000-39,999 Baht, Rural 15,000-29,999 Baht)  C (กทม. 30,000-49,999 บาท , Urban 20,000-39,999 บาท, Rural 15,000-29,999 บาท) | 3 |  |
|  | D (GBKK 20,000-29,999 Baht, Urban 15,000-19,999 Baht, Rural 10,000-14,999 Baht)  D (กทม. 20,000-29,999 บาท , Urban 15,000-19,999 บาท, Rural 10,000-14,999 บาท) | 4 |  |
|  | E (GBKK <20,000 Baht, Urban <15,000 Baht, Rural <10,000 Baht)  E (กทม.<20,000 บาท, Urban <15,000 บาท, Rural <10,000 บาท) | 5 |  |

**Malaysia**

| Q13a | **QUESTION**  **PROGRAMMER NOTE:**  **ASK ALL**  Which of the following income categories best describes your **monthly household income** from all sources? (Household income refers to the combined incomes of all members of your household. Income includes salary/ wages, retirement and state benefits, returns from investments, scholarship funds, allowances etc) [SA] |
| --- | --- |
| Q13b | **PROGRAMMER NOTE:**  **Q13B VALUE CANNOT BE GREATER THAN Q13A VALUE**  **ASK ALL**  Which of the following best describes your **monthly personal income** from all sources? (Personal Income refers to your own total income. Income includes salary/ wages, retirement and state benefits, returns from investment, scholarship funds, allowances, etc) [SA] |

|  |  | Q13a | Q13b |
| --- | --- | --- | --- |
|  |  | (H1)  Monthly Household Income | (H1)  Monthly Personal Income |
|  |  | (173-174) | (175-176) |
| (R1) | Up to RM 1,000 | 01 | 01 |
| (R2) | RM1,001-RM1,500 | 02 | 02 |
| (R3) | RM1,501-RM2,000 | 03 | 03 |
| (R4) | RM2,001-RM2,500 | 04 | 04 |
| (R5) | RM2,501-RM3,000 | 05 | 05 |
| (R6) | RM3,001-RM3,500 | 06 | 06 |
| (R7) | RM3,501-RM4,000 | 07 | 07 |
| (R8) | RM4,001-RM5,000 | 08 | 08 |
| (R9) | RM5,001 - RM5,500 | 09 | 09 |
| (R10) | RM5,501 - RM6,000 | 10 | 10 |
| (R11) | RM6,001 - RM6,500 | 11 | 11 |
| (R12) | RM6,501 - RM7,000 | 12 | 12 |
| (R13) | RM7,001 - RM7,500 | 13 | 13 |
| (R14) | RM7,501 - RM8,000 | 14 | 14 |
| (R15) | RM8,001 - RM8,500 | 15 | 15 |
| (R16) | RM8,501 - RM9,000 | 16 | 16 |
| (R17) | RM9,001 - RM9,500 | 17 | 17 |
| (R18) | RM9,501 - RM10,000 | 18 | 18 |
| (R19) | RM10,001 - RM15,000 | 19 | 19 |
| (R20) | Above RM15,000 | 20 | 20 |
| (R99) | Not disclosed/refused (DO NOT SHOW/READ) | CLOSE | CLOSE |

| Q13c | **PROGRAMMER NOTE:**  **HIDE QUESTION FROM LIVE LINK**  **AUTOCODE 1 IF Q13A = 9-20**  **AUTOCODE 2 IF Q13A = 6-8**  **AUTOCODE 3 IF Q13A = 4-5**  **AUTOCODE 4 IF Q13A = 2-3**  **AUTOCODE 5 IF Q13A = 1**  Record MHI SEC[SA] | Code  (130) | Route |
| --- | --- | --- | --- |
|  | SEC A (RM5,001 and above) | 1 |  |
|  | SEC B (RM3,001 - RM5,000) | 2 |  |
|  | SEC C (RM2,001 - RM3,000) | 3 |  |
|  | SEC D (RM1,001 - RM2,000) | 4 |  |
|  | SEC E (Below RM1,001) | 5 |  |

**Thank you for your time and responses to this survey.**

1. COMMT Partners are – Alive & Thrive, Jane Badham Consulting, HKIs ARCH Project, Access to Nutrition Initiative (ATNI), WFP Regional Office for Asia and Pacific, WHO SEARO (observer only) and UNICEF EAPRO. [↑](#footnote-ref-2)
2. Definition of Packaged Complementary Food (aka CACF): Commercially available complementary foods (CACF) are all commercially available foods and beverages that are specifically marketed as suitable for feeding older infants and young children if they meet at least one of the following criteria:  **1.** are recommended for introduction at an age of less than 3 years**;  2**. are labelled with the word’s ’baby’, ’toddler’, ’young child’, or synonym;  **3**. have a label with an image of a child who appears to be younger than 3 years of age or who is feeding with a bottle; or **4.** are in any other way presented as being suitable for children under the age of 3 years. (WHO, 2016; WHO, 2019).   [↑](#footnote-ref-3)
3. Definition of Packaged Complementary Food (aka CACF): Commercially available complementary foods (CACF) are all commercially available foods and beverages that are specifically marketed as suitable for feeding older infants and young children if they meet at least one of the following criteria:  **1.** are recommended for introduction at an age of less than 3 years**;  2**. are labelled with the word’s ’baby’, ’toddler’, ’young child’, or synonym;  **3**. have a label with an image of a child who appears to be younger than 3 years of age or who is feeding with a bottle; or **4.** are in any other way presented as being suitable for children under the age of 3 years. (WHO, 2016; WHO, 2019).   [↑](#footnote-ref-4)
4. Questions L5 and L6 based on From FDA Food Safety and Nutrition Survey https://www.fda.gov/media/146532/download [↑](#footnote-ref-5)
